# Metacognition and mentalizing are associated with distinct neural representations of decision uncertainty

**DOI:** 10.1371/journal.pbio.3001301

**Published:** 2022-05-13

**Authors:** Shaohan Jiang, Sidong Wang, Xiaohong Wan

**Affiliations:** State Key Laboratory of Cognitive Neuroscience and Learning and IDG/McGovern Institute for Brain Research, Beijing Normal University, Beijing, China; Oxford University, UNITED KINGDOM

## Abstract

Metacognition and mentalizing are both associated with meta-level mental state representations. Conventionally, metacognition refers to monitoring one’s own cognitive processes, while mentalizing refers to monitoring others’ cognitive processes. However, this self-other dichotomy is insufficient to delineate the 2 high-level mental processes. We here used functional magnetic resonance imaging (fMRI) to systematically investigate the neural representations of different levels of decision uncertainty in monitoring different targets (the current self, the past self [PS], and others) performing a perceptual decision-making task. Our results reveal diverse formats of internal mental state representations of decision uncertainty in mentalizing, separate from the associations with external cue information. External cue information was commonly represented in the right inferior parietal lobe (IPL) across the mentalizing tasks. However, the internal mental states of decision uncertainty attributed to others were uniquely represented in the dorsomedial prefrontal cortex (dmPFC), rather than the temporoparietal junction (TPJ) that also represented the object-level mental states of decision inaccuracy attributed to others. Further, the object-level and meta-level mental states of decision uncertainty, when attributed to the PS, were represented in the precuneus and the lateral frontopolar cortex (lFPC), respectively. In contrast, the dorsal anterior cingulate cortex (dACC) represented currently experienced decision uncertainty in metacognition, and also uncertainty about the estimated decision uncertainty (estimate uncertainty), but not the estimated decision uncertainty per se in mentalizing. Hence, our findings identify neural signatures to clearly delineate metacognition and mentalizing and further imply distinct neural computations on internal mental states of decision uncertainty during metacognition and mentalizing.

## Abstract 2

The relationship between metacognition and mentalizing is still a matter of debate, as both are associated with meta-representations. This study adapts a task paradigm used in metacognition to apply in mentalizing and compares the neural representations of decision uncertainty in metacognition and mentalizing.

## Introduction

Humans are social beings. We interact with others not only in the physical world but also in the mental world. Differing from objects in the physical world, humans are free and intentional agents who hold mental states that are not necessarily reflections of reality in the physical world. If there are a thousand readers, there must be a thousand Hamlets. Even though the physical world is same, the readers’ mental worlds are different from one another. The human brain thus needs to concurrently represent different mental states in the mental worlds of both the self and others during social interactions [1–3]. Failures of normal development of such an ability may cause deficits in human cognition and behaviors, e.g., in autism spectrum disorder (ASD) and schizophrenia [4,5]. Thus, it is a central question in psychology and neuroscience to understand the mechanisms of human mental state representations.

A principal criterion conventionally used to distinguish nonsocial activities from social activities is whether the activities are conducted toward the self or others [6–9]. A corresponding distinction is also drawn on mental state attributing processes: Monitoring one’s own cognitive processes is referred to as metacognition [10], but when the target participant is an intentional agent other than the self, it is referred to as mentalizing [11–13]. Although both metacognition and mentalizing involve meta-representations of the mental worlds [1–3], the representational formats and the sources of the mental states differ. Critically, mentalizing necessitates others’ perspective taking to infer their mental states [14], while one’s own mental states are directly accessible by inspection [1,2].

**Fig 1:**
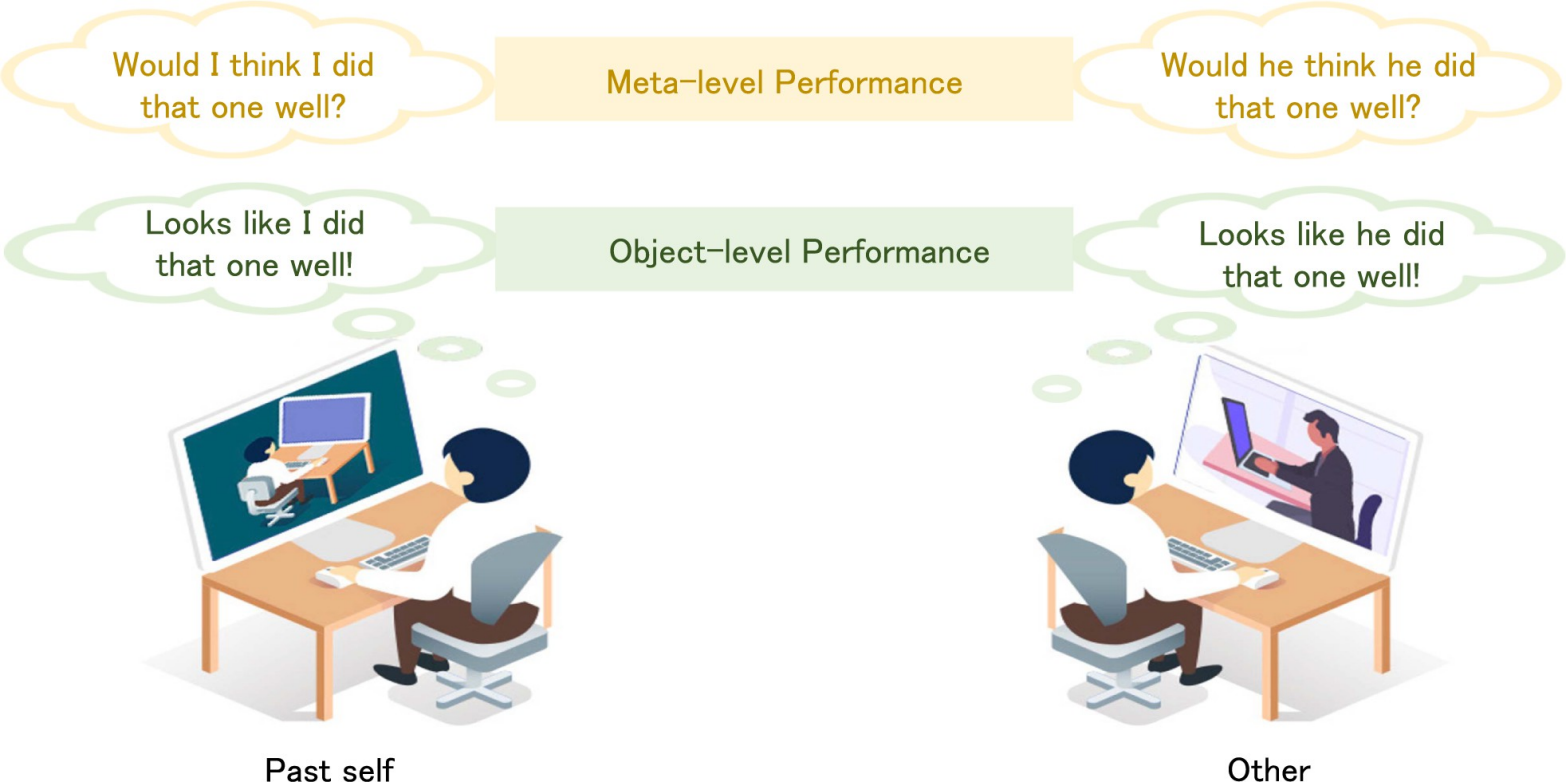
The meta-representations in mentalizing under different social contexts. The observer often needs to estimate others’ covert cognitive states from external cue information during social interactions, e.g., to infer others’ decision uncertainty by observing their task performance. According to the framework of metacognition, others’ cognitive states can be hierarchically divided into meta-level and object-level states: The metal-level states reflect internal monitor on their object-level states. Accordingly, the mentalizing processes in monitoring others’ object-level and meta-level performance might be differential (right side). Occasionally, we may estimate the PS momentary cognitive states (left side). The mental state representations might be different in attributing different cognitive states to different target participants. PS, past self.

However, the self-other dichotomy on the target agents is insufficient to discern the 2 processes. First, similar to attributing mental states to others, the momentary mental states of the past self (PS) cannot be concurrently experienced by inspection as in metacognition, but are inferred from the available external cue information (e.g., facial expressions) or from the episodic memory cued by the external information. It thus becomes ambiguous whether such mental state representations in attributing to the PS should be classified as metacognition or mentalizing (Fig 1). Second, one’s own mental states are hierarchically categorized into type 1 (object level) and type 2 (meta level) mental states in metacognition [10]. The meta-level mental states about the mental world are the signals generated during monitoring the object-level mental states in response to the physical world. For example, the belief (meta level) about whether one’s decision is correct (object level). Accordingly, the mental states attributed to others in mentalizing could also be hierarchically categorized into 2 levels concerned with object-level and meta-level performance, respectively (Fig 1). Thereby, the representations of the 2-level mental states even attributed to the same target in mentalizing might be different. Third, in attributing the object-level mental states to others or the PS, the meta-level mental states are actually generated by the observer, rather than the target participants. In this sense, the meta-level mental state representations appear more similar to those in metacognition than mentalizing. Hence, the distinctions between metacognition and mentalizing along the self-other dichotomy remain considerably ambiguous.

We then look to neural signatures to delineate metacognition and mentalizing. Surprisingly, although a number of disparate studies on the neural mechanisms of metacognition and mentalizing have been conducted in cognitive neuroscience [15–17] and social neuroscience [18,19], respectively, a direct comparison of the 2 neural processes is so far lacking. This unusual situation might be primarily due to the lack of an appropriate experimental paradigm applicable for both processes. The mental state that is mainly concerned in studies of metacognition is decision uncertainty or decision confidence. Decision uncertainty is the opposite of decision confidence where decision confidence is a belief about that one’s own decision is correct. Decision uncertainty serves as a control signal to improve one’s decision even with no external feedback [15,20]. If a higher level of decision uncertainty is retrospectively monitored, then more cognitive control is consequently evoked. On the other hand, decision uncertainty also serves as a critical social control signal for efficacious decision improvement in joint decision-making [21,22]. Hence, it is of great importance to understand mental state representations of decision uncertainty in metacognition and mentalizing.

Inferences of others’ mental states in mentalizing are often made under social contexts with external cue information. The mental states attributed to others might be inferred through object-level associations between external cue information and covert mental states. For example, inferring decision uncertainty from others’ hesitations in responses (i.e., reaction times), rather than on the basis of others’ metacognitive abilities. Associating external cue information with covert mental states may also lead to predict others’ performance. Thereby, it is difficult to discern the underlying cognitive processes merely from the observed behaviors [23–26]. Because of this ambiguity, to date, it remains unclear whether or not nonhuman primates can mentalize, namely creating a mental model simulating others’ mental world and generating internal mental state representations (i.e., theory of mind, ToM) [23–26]. To demonstrate that this mentalizing capability exists in humans or animals, one approach is to identify internal mental state representations that is unassociated with external cue information. Although the neural correlates of external cue information might not merely comprise the cue associations, the existence of neural signatures of internal mental state representations should undoubtedly endorse mentalizing.

In the current study, we aimed to delineate the neural representations of decision uncertainty attributed to different target participants: the current self, the PS, and others. To do so, we adapted a task paradigm often used in metacognition to apply to mentalizing. By means of such task alignments, we could compare the 2 mental processes in a similar task context. Further, we could compare the different mentalizing processes in the same task context, to specify whether the mental state representations are shared or segregated in attributing to different targets. That is, whether it is the target that matters (different neural signatures despite of similar computations) or the computation that matters (same neural signatures despite of different targets). We segregated decision uncertainty into 2 dissociated components—associations with external cue information and internal mental states unassociated with external cue information. We took the residuals after regressing out external cue information from decision uncertainty reported by the participants as a proxy of internal mental states in each task. We used functional magnetic resonance imaging (fMRI) to separately characterize the neural correlates of external cue information and residuals in attributing decision uncertainty to others and the PS in mentalizing and to the current self in metacognition. Our results reveal diverse representations of internal mental states in the mentalizing tasks, but a general format of internal mental state representations in metacognition.

## Results

### Task paradigm

**Fig 2:**
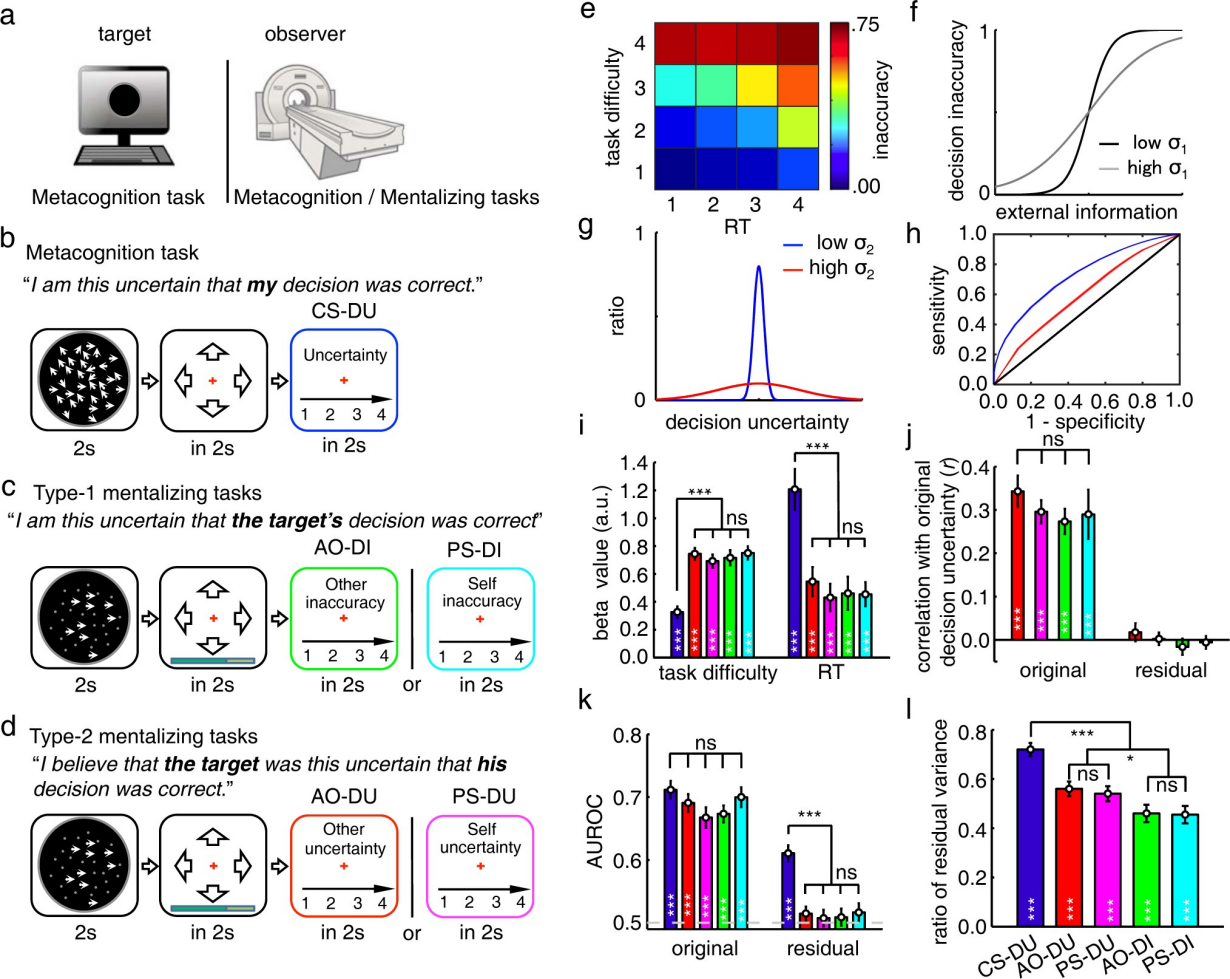
Task paradigms and behavioral results. (**a**) The fMRI experimental setup. During the metacognition task, the participant inside the MRI scanner did the metacognition task alone. During the mentalizing tasks, the participant inside the MRI scanner observed the task performance on the metacognition task done by the target participant who was outside the scanner. (**b**) The metacognition task: The participant completed the RDM task and reported his/her CS-DU. (**c**) The type 1 mentalizing tasks: The participant observed the RDM task performance by a target participant and reported the target participant’s decision inaccuracy. The target participant was either an AO-DI or the PS-DI. (**d**) The type 2 mentalizing task: Instead of judging the target participants’ decision inaccuracy, the participants estimated decision uncertainty that would be concurrently reported by the AO/PS in the current trial (AO-DU/PS-DU). (**e**) The decision inaccuracy changed with task difficulty and RT in the AO-DU task, averaged across participants (n = 28). (**f**) Theoretically, decision inaccuracy is a sigmoid function of task difficulty and RT on each trial. Each target participant has unique internal noise (*σ*_1_) in perceptual decision-making that causes different variances in decision inaccuracy. (**g**) Theoretically, in estimating the target participant’s decision uncertainty, the unique internal noise (*σ*_2_) needs to be further considered in mapping decision inaccuracy to decision uncertainty. (**h**) Theoretically, different levels of internal noise (*σ*_2_) cause different metacognitive abilities (AUROC). (**i**) The regression beta values of the normalized (task) difficulty and RT with the estimates in each task. The weights did not differ across mentalizing tasks (ANOVA, task difficulty: F_[3,112]_ = 0.11, *P* = 0.95; RT: *F*_[3,112]_ = 0.16, *P* = 0.92), but significantly differ from the metacognition task (2-tailed paired *t* test, task difficulty: t_27_ = 6.2; *P* = 2.5 × 10^−8^; RT: t_27_ = 4.1; *P* = 5.9 × 10^−5^). (**j**) The correlation between the estimated decision inaccuracy/uncertainty in the mentalizing tasks with the target participant’s actual decision uncertainty reported in the metacognition task, before (original: ANOVA, F_[3,112]_ = 0.28, *P* = 0.84) and after (residual: *P*s > 0.30) the associations with external cue information were regressed out. (**k**) The consistency between the estimated decision inaccuracy/uncertainty and the actual decision outcome (true or false) measured by the AUROC, before (original: ANOVA, F_[4,139]_ = 1.22, *P* = 0.31) and after (residual: *P*s > 0.20) the associations with external cue information were regressed out. (**l**) The ratio of estimate residuals to the total estimate variances (2-tailed *t* test, AO: t_27_ = 2.5; *P* = 0.0096 in the contrast between the type 2 and type 1 mentalizing tasks; PS: t_27_ = 2.1; *P* = 0.023 in the contrast between the type 2 and type 1 mentalizing tasks; 2-tailed paired *t* test, t_27_ = 3.5; *P* = 3.3 × 10^−4^ in the contrast between the metacognition task and the mentalizing tasks). The error bars represent SEM across participants. *P < 0.05; **P < 0.01; ***P < 0.001, after Bonferroni correction. The raw data for Fig 2 can be found in the Supporting information as S1 Data. ANOVA, analysis of variance; AO-DI, anonymous other decision inaccuracy; AUROC, area under the ROC curve; CS-DU, current-self decision uncertainty; fMRI, functional magnetic resonance imaging; PS-DI, past-self decision inaccuracy; RDM, random dots motion; RT, reaction time; SEM, standard error of the mean.

We carried out 3 fMRI experiments to investigate the mental state representations of decision uncertainty in metacognition and mentalizing (Fig 2A; S1 Fig). A total of 28 healthy participants took part in all of the 3 experiments (see Methods). In experiment 1, each participant judged the gross motion direction of random moving dots and rated his/her uncertainty about the preceding decision (Fig 2B). There were 4 different task difficulty levels randomly mixed in the task (S2 Fig). Hereafter, this perceptual decision-making task was referred to as the random dots motion (RDM) task (Fig 2A). The participant reported the current-self decision uncertainty (CS-DU), immediately accompanying perceptual decision-making in each trial.

In experiment 2, the participant inside the scanner observed an anonymous other (AO) concurrently performing the RDM task outside the scanner and judged the AO’s decision inaccuracy (AO-DI). Decision inaccuracy is the opposite of decision accuracy where decision accuracy is the objective probability that the AO’s decision is correct (Fig 2C). Differing from the metacognition task (CS-DU), the partner’s cognitive states were inaccessible. It thus necessitated the participant to infer the probability that the partner’s decision was correct. To avoid evoking the participant’s own decision uncertainty, the stimuli presented to the participant were noiseless: Only coherently moving dots were moving, whereas randomly moving dots remained stationary. By virtue of this altered stimulus presentation, the participant could perceive the task difficulty without evoking his/her own decision uncertainty (Fig 2C). This is a necessary condition to dissociate the neural representations of decision uncertainty in mentalizing from those in metacognition. Otherwise, the participant might use his/her own decision uncertainty to estimate the partner’s decision uncertainty. The partner’s response time (RT) was reported to the participant by a progress color bar, whereas neither the choice nor the reported decision uncertainty by the partner was presented to the participant. Hence, the participant could only use the external cue information of task difficulty and RT to estimate the partner’s decision inaccuracy. In a parallel task, the participant instead observed task performance on the metacognition task done past by himself/herself and judged the past-self decision inaccuracy (PS-DI). Otherwise, the experimental procedure was identical to the AO-DI task. Notably, as the past decision-making processes by oneself were also inaccessible and the past mental states associated with similar stimuli were impossible to explicitly memorize, the underlying cognitive process might also be mentalizing. We therefore refer to the 2 tasks as the type 1 mentalizing tasks.

In experiment 3 (Fig 2D), the experimental procedure was identical to experiment 2, but the participant estimated the target participants’ mental states of decision uncertainty in each trial, namely the participant estimated the target participants’ believes about whether their own decisions were correct. The 2 tasks thus also entailed mentalizing to attribute type 2 mental states of decision uncertainty to the AO/PS (AO-DU and PS-DU). We therefore refer to the 2 tasks as the type 2 mentalizing tasks.

Experiment 1 and experiment 3 were conducted in the same session, but experiment 2 was conducted in another session. To reduce confusion between type 1 and type 2 mentalizing tasks, the 2 sessions were separated at least over 2 weeks. The tasks in each session were randomly interleaved and were counterbalanced across the participants.

The task sequences of the 4 mentalizing tasks were identical, only the instructions differed. Thereby, any behavioral and neural differences between them should be caused by different mentalizing processes. The task sequences of the metacognition task and the mentalizing tasks were also quite similar. However, the differences between the 2 types of tasks existed in both the perception phase and the judgment phase. Here, however, we are not so much concerned with the former but with the latter phase. In particular, the participant currently experienced decision uncertainty accompanying perceptual decision-making in the metacognition task, but inferred decision uncertainty that was not concurrently experienced from the cue information in the mentalizing tasks. However, because the perception phase and the judgment phase were temporally close to each other, it might be argued that any difference in neural signatures is due to the difference in the stimulus presentation as opposed to the difference between the metacognitive and metalizing processes. To confirm whether the neural correlates between the 2 phases are separable, we made analyses on the simulated fMRI signals generated by the same task sequence. Our simulation analyses demonstrated that the neural correlates of decision uncertainty in the perception phase or the judgment phase could be dissociated by conventional general linear models (GLMs) (S3A Fig).

### Hierarchical mental state representations of decision inaccuracy and decision uncertainty in mentalizing

To assess behavioral metrics used for data analyses, we made theoretical analyses on mental state representations of decision inaccuracy and decision uncertainty in mentalizing. According to the decision-making theory [27], decision inaccuracy is crucially dependent on both task difficulty and RT. The higher the task difficulty and the longer the RT, the higher the decision inaccuracy (Fig 2E). For the sake of simplicity, decision inaccuracy is assumed to be a sigmoid function of task difficulty and RT (Eq 1 in Methods; Fig 2F). Hence, it is plausible to estimate decision inaccuracy and decision uncertainty in the mentalizing tasks merely from external information provided by task difficulty and RT. However, one indispensable process to distinguish social inferences in mentalizing from nonsocial inferences or associations is taking the target participant’s perspective. For example, in the type 1 mentalizing tasks, the participants should consider that the target participant has unique internal noise (*σ*_1_) during the perceptual decision-making process as described by the drift-diffusion model [27], which affects the target participant’s object-level performance (i.e., decision inaccuracy, Fig 2F). In the type 2 mentalizing tasks, the participant should further consider that the target participant has unique internal noise (*σ*_2_) in mapping decision inaccuracy to decision uncertainty (Fig 2G), which renders the target participant’s unique metacognitive ability even with the same object-level performance [28] (i.e., a low variance results in a high metacognitive ability). We constructed the receiver operating characteristic (ROC) curve by using the level of decision uncertainty as the criterion to judge the incorrectness of the choice in each trial and measured the metacognitive ability as the area under the ROC curve (AUROC), indicating the extent to which the subjective uncertainty ratings matched the actual decision inaccuracy [28] (Fig 2H). Taken together, the internal mental state representations of decision inaccuracy and decision uncertainty in mentalizing should be hierarchically organized. However, due to the lack of feedback in the mentalizing tasks, each participant did not learn about the target participants’ (even for the PS) object-level and meta-level performance. Therefore, the internal information generated by mentalizing may not reflect the target participant’s actual internal mental states.

### Behavioral results

We analyzed the behavioral data in the experiments to assess how the participants used the associations with external cue information of task difficulty and RT to estimate the targets’ decision inaccuracy and decision uncertainty. The analyses showed that the weights of normalized task difficulty and RT (Eq 1 in Methods) on the estimates were equivalent [analysis of variance (ANOVA), task difficulty: F_[3,112]_ = 0.11, *P* = 0.95; RT: F_[3,112]_ = 0.16, *P* = 0.92; Fig 2I] and were highly correlated across the mentalizing tasks (S4 Fig). Hence, the participants used such external cue information to estimate the corresponding mental states equally across the mentalizing tasks. Notably, the estimates in the mentalizing tasks relied more on task difficulty than RT (2-tailed paired *t* test, t_27_ = 6.2; *P* = 2.5 × 10^−8^). On the contrary, the estimates of decision uncertainty in the metacognition task relied more on RT than task difficulty (2-tailed paired *t* test, t_27_ = 4.1; *P* = 5.9 × 10^−5^; Fig 2I). This is likely due to the fact that stimulus coherence (by virtue of the nature of the experimental design) was clearly discerned in the mentalizing tasks, but it was hard to inversely infer the stimulus coherences in the metacognition task. Because of the stable associations with external cue information, the decision inaccuracy/uncertainty estimated by the participants in the mentalizing tasks was correlated with what the target participants had actually reported themselves at each level of difficulty and RT in the metacognition task (ANOVA, F_[3,112]_ = 0.28, *P* = 0.84, Fig 2J).

However, all these correlations in the mentalizing tasks disappeared after regressing out the associations with external cue information of task difficulty and RT from the estimates of decision inaccuracy/uncertainty reported by the participants (2-tailed *t* test, *P*s > 0.30; Fig 2J). That is, estimate residuals in each mentalizing task did not further predict the actual decision uncertainty reported by the target participants in the metacognition task. Further, as the estimates of decision uncertainty in each task could largely predict the actual decision inaccuracy, we used the AUROC to characterize this consistency. On average, the AUROCs (mean: 0.67 to 0.71) were larger than the chance level that was calculated by shuffling the orders between the estimates and actual decision inaccuracy and showed no significant differences across all the tasks (ANOVA, F_[4,139]_ = 1.22, *P* = 0.31, Fig 2K). After the associations with external cue information were regressed out, the residual AUROCs (measured by estimate residuals) were no longer significantly different from the chance level in each mentalizing task (2-tailed *t* test, *P*s > 0.20), but it remained significant in the metacognition task (2-tailed *t* test, t_27_ = 11.8; *P* = 3.6 × 10^−12^; Fig 2K). Estimate residuals in the metacognition task should reflect the subjective difficulty due to the trial-by-trial noises in internal neural processing that task difficulty (coherence) and RT could not explain [29]. Thereby, the residuals also considerably contributed to decision uncertainty in the metacognition task. In striking contrast, reliable estimates of decision inaccuracy/uncertainty in the mentalizing tasks were crucially dependent on external information provided by task difficulty and RT.

Nonetheless, estimate residuals accounted for about half of the total variance of the estimates of decision inaccuracy/uncertainty in each mentalizing task (Fig 2L), although each ratio was much lower than that in the metacognition task (2-tailed paired *t* test, t_27_ = 3.5; *P* = 3.3 × 10^−4^; Fig 2L). These estimate residuals might serve as a proxy of internal mental states that were generated through target participant’s perspective taking and were independent of external cue information. We thus used estimate residuals as the main behavioral metric to identify the neural representations of internal mental states. As indirect evidence, the variances of estimate residuals were significantly larger in the type 2 mentalizing tasks than the type 1 mentalizing tasks (2-tailed *t* test, AO: t_27_ = 2.5; *P* = 0.0096; PS: t_27_ = 2.1; *P* = 0.023). These extra variances might be generated by the additional process of the target participant’s meta-level perspective taking in the type 2 mentalizing tasks, as suggested by the hierarchical mental state representation model described above (Fig 2G and 2H).

### Common neural representations of external cue information in mentalizing

**Fig 3:**
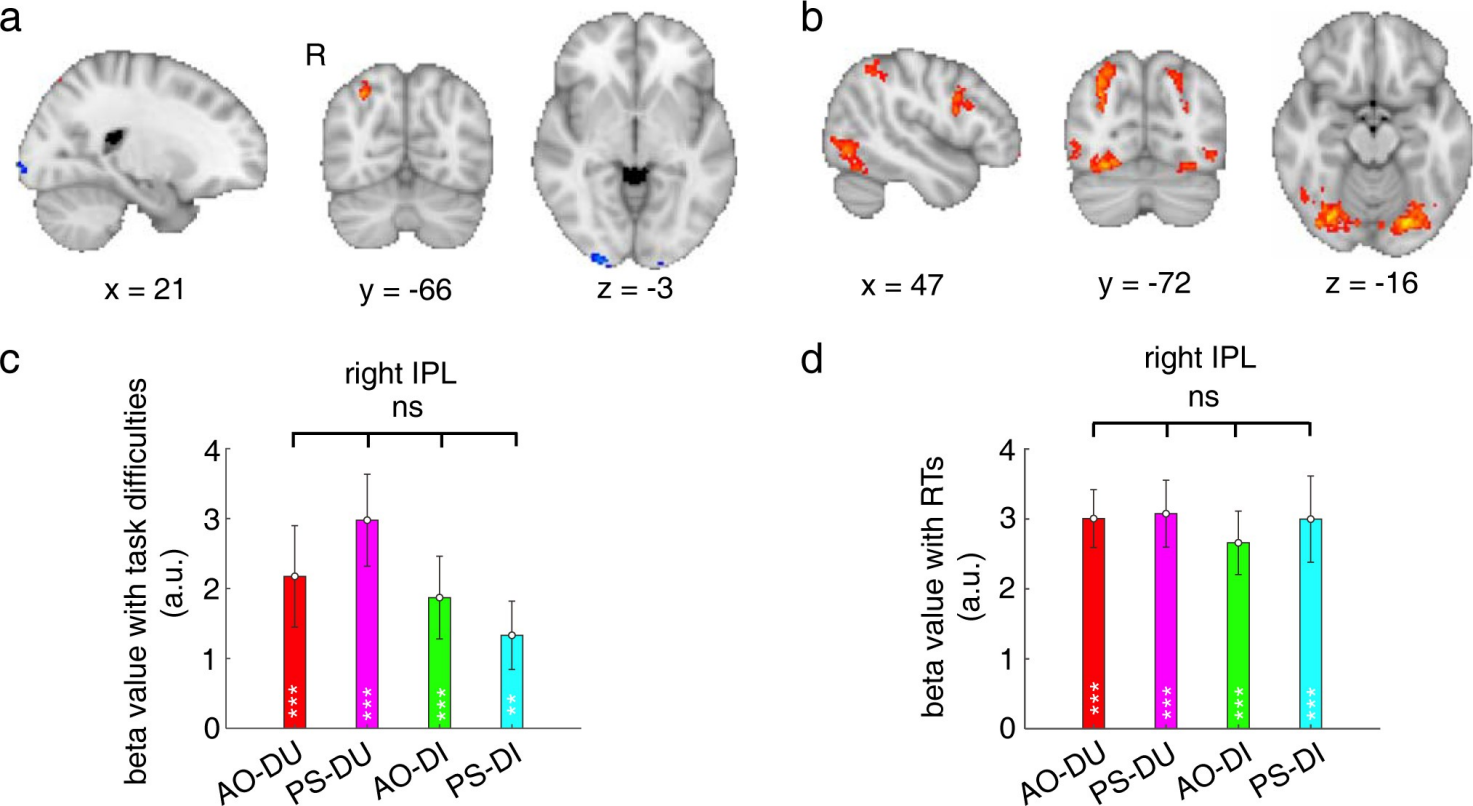
Common neural representations of external cue information across the mentalizing tasks. (**a**) The activation maps for the activities significantly correlated with task difficulty in a conjunction analysis across the mentalizing tasks (*z* > 2.6, *P* < 0.05 after cluster-level FWE correction). (**b**) The activation maps for the activities significantly correlated with RT in a conjunction analysis across the mentalizing tasks (*z* > 2.6, *P* < 0.05 after cluster-level FWE correction). The activation maps in (**a**) and (**b**) were displayed in radiological convention (the left/right side of the image corresponds to the right/left side of the brain). (**c**) The parametric regression beta values of task difficulty in each mentalizing task in the right IPL ROI defined by the conjunction of (**a**) and (**b**). (**d**) The beta values of RT in each mentalizing task in the right IPL ROI defined by the conjunction analysis of (**a**) and (**b**). The error bars represent SEM across participants. *ns*, not significant; *P < 0.05; **P < 0.01; ***P < 0.001, uncorrected. The raw data for Fig 3 can be found in the Supporting information as S1 Data. AO-DI, anonymous other decision inaccuracy; AO-DU, anonymous other decision uncertainty; FWE, family-wise error; IPL, inferior parietal lobe; PS-DI, past-self decision inaccuracy; PS-DU, past-self decision uncertainty; ROI, region of interest; RT, response time; SEM, standard error of the mean.

We first examined the neural representations of external cue information in mentalizing by analyzing the fMRI data acquired during the experiments. As external cue information of task difficulty and RT contributed to the estimates equally across the mentalizing tasks, we hypothesized that the neural representations of each type of external cue information might be shared across the mentalizing tasks. To test the hypothesis, we regressed the trial-by-trial fMRI activities during the judgment phase with external cue information of task difficulty and RT across the whole brain in each task (see Methods). Across the mentalizing tasks, the fMRI activities in the primary visual cortex (V1) were negatively correlated with the levels of task difficulty (the conjunction analysis, *z* > 2.6, *P* < 0.05 after cluster-level family-wise error (FWE) correction; Fig 3A), decreasing as the number of moving dots was reduced (task difficulty increased). On the other hand, the fMRI activities in the right inferior parietal lobe (IPL) were positively correlated with the levels of task difficulty (the conjunction analysis, *z* > 2.6, *P* < 0.05 after cluster-level FWE correction; Fig 3A). In contrast, the fMRI activities in a wide range of brain regions were positively correlated with the levels of RTs (the conjunction analysis, *z* > 2.6, *P* < 0.05 after cluster-level FWE correction; Fig 3B). Among these brain regions, the right IPL region overlapped with the regions associated with task difficulty: The same right IPL region responded to both task difficulty and RT across the mentalizing tasks (Fig 3C and 3D). Thus, integration of the 2 pieces of external information together in the right IPL partially contributed to the estimates of decision inaccuracy/uncertainty in mentalizing.

### Distinct neural representations of estimate residuals in mentalizing

We then examined the neural representations of internal mental states that were unassociated with external cue information in mentalizing. According to our theoretical analyses as described above, additional unique processes of target participant’s perspective taking should be involved in each mentalizing task besides the associations with external cue information. To explore the neural signatures, we regressed estimate residuals with the trial-by-trial fMRI activities during the judgment phase across the whole-brain voxels in each task. These neural correlates thus illustrated internal mental state representations that were unassociated with external information. The modulation effects were predominately in the judgment phase by comparison to the alternative GLM accounting for the modulation effects in the perception phase (S3B Fig).

**Fig 4:**
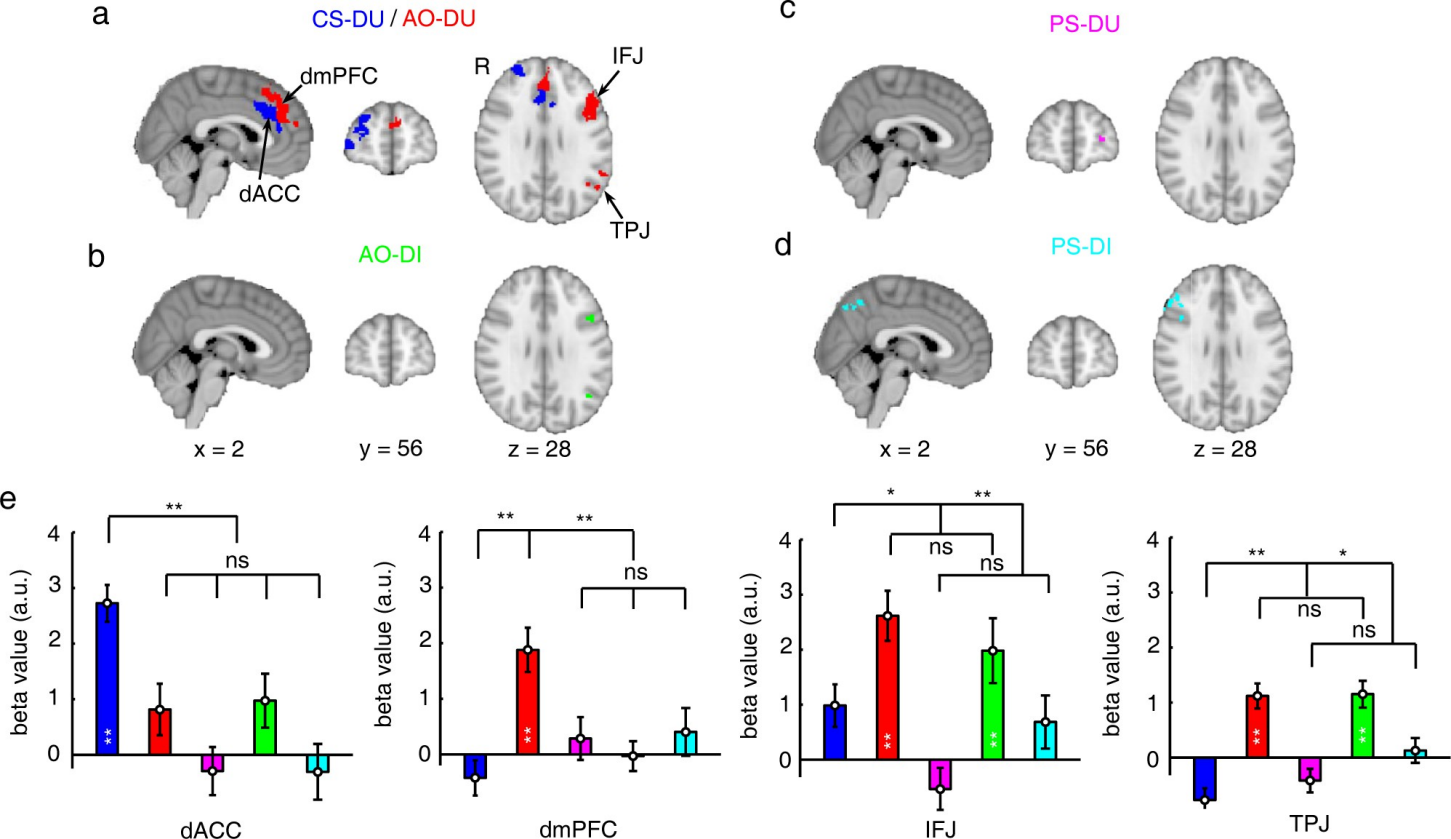
Distinct neural representations of estimate residuals in metacognition and mentalizing. (**a**) Neural correlates of estimate residuals of decision uncertainty in the metacognition task (dACC and lFPC, blue) and neural correlates of estimate residuals of decision uncertainty in the AO-DU (type 2) mentalizing task (dmPFC, left IFJ and left TPJ, red). (**b**) Neural correlates of estimate residuals of decision inaccuracy in the AO-DI (type 1) mentalizing task (left IFJ and left TPJ, green). (**c**) Neural correlates of estimate residuals of decision uncertainty in the PS-DU (type 2) mentalizing task (left lFPC, magenta). (**d**) Neural correlates of estimate residuals of decision inaccuracy in the PS-DI (type 1) mentalizing task (right-IFJ and precuneus, cyan). All the activation maps were displayed in radiological convention. (**e**) The comparisons of the parametric regression beta values with estimate residuals across tasks in the ROIs of dACC, dmPFC, left IFJ, and left TPJ (see also S5 Fig). The error bars represent SEM across participants. *ns*, not significant; *P < 0.05; **P < 0.01 after Bonferroni correction. The raw data for Fig 4 can be found in the Supporting information as S1 Data. AO-DI, anonymous other decision inaccuracy; AO-DU, anonymous other decision uncertainty; CS-DU, current-self decision uncertainty; dACC, dorsal anterior cingulate cortex; dmPFC, dorsomedial prefrontal cortex; IFJ, inferior frontal junction; lFPC, lateral frontopolar cortex; PS-DI, past-self decision inaccuracy; PS-DU, past-self decision uncertainty; ROI, region of interest; SEM, standard error of the mean; TPJ, temporoparietal junction.

When the participant estimated the AO’s decision uncertainty, estimate residuals were significantly correlated with the fMRI activities in the dorsomedial prefrontal cortex (dmPFC; red in Fig 4A), dorsally and anteriorly neighboring, but separate from, the dorsal anterior cingulate cortex (dACC) region representing the CS-DU in metacognition (blue in Fig 4A; see below), as well as in the left temporoparietal junction (TPJ) and the left inferior frontal junction (IFJ) (*z* > 3.1, *P* < 0.05 after cluster-level FWE correction, Fig 4A; see also S1 Table).

When the participant estimated the AO’s decision inaccuracy, estimate residuals were also correlated with the fMRI activities in the left TPJ and the left IFJ (*z* > 3.1, *P* < 0.05 after cluster-level FWE correction, Fig 4B and 4E; see also S1 Table), but not in the dmPFC (2-tailed *t* test, t_27_ = 1.1, *P* = 0.12; Fig 4E).

The dmPFC was selectively involved in type 2 mentalizing (Fig 4E; the post hoc comparisons with the other tasks: *P* < 0.0037 after Bonferroni correction), but the TPJ was involved in both type 1 and type 2 mentalizing (Fig 4E; 2-tailed paired *t* test, t_27_ = 0.67, *P* = 0.21 between the 2 mentalizing tasks, the post hoc comparisons with the other tasks: *P* < 0.042 after Bonferroni correction). To further test the reliability of the dmPFC selectivity in type 2 mentalizing, we repeated the same GLM analysis on the dmPFC and TPJ regions independently defined by meta-analytical maps from the NeuroSynth database [30], as well as the conjunction regions between the meta-analytical regions and those in the current study. In both analyses, the results consistently support that the dmPFC but not the TPJ showed neural activities selective to type 2 mentalizing (S5 Fig).

Instead, estimate residuals of the past-self decision uncertainty (PS-DU) were selectively correlated with the fMRI activities in the left lateral frontopolar cortex (lFPC) (*z* > 3.1, *P* < 0.05 after cluster-level FWE correction, Fig 4C; but not significantly different from the other tasks, S6F Fig), whereas estimate residuals of the PS-DI were correlated with the fMRI activities in the precuneus (*z* > 3.1, *P* < 0.05 after cluster-level FWE correction, Fig 4D; the comparisons with the other tasks: *P* < 0.043 after Bonferroni correction, S6E Fig). Notably, both the left lFPC and precuneus regions were also shared with the metacognition-associated areas (S6E and S6F Fig).

In accordance with our theoretical account, the existence of different neural correlates of estimate residuals in the mentalizing tasks suggested that there are diverse neural representations of internal mental states during mentalizing in different social contexts. Notably, the right IPL that encoded task difficulty and RT did not represent estimate residuals in any of the mentalizing tasks (S6A Fig). The neural representations of internal mental states suggest that internal mental state representations do separately coexist with external cue associations during mentalizing (attributing covert mental states to the other target participants) at least, in humans. This was, however, could not have been inferred from behavioral observation alone.

### Neural representations of estimate residuals in metacognition

In the metacognition task, estimate residuals were significantly correlated with the fMRI activities in the dACC and the lFPC (*z* > 3.1, *P* < 0.05 after cluster-level FWE correction, blue in Fig 4A; see also S1 Table), as repeatedly observed in previous studies [15–17,31,32]. Although the lFPC region was also associated with type 2 mentalizing (S6F Fig), the dACC region selectively represented estimate residuals in the metacognition task, but not in the mentalizing tasks (S6B Fig; the post hoc comparisons between the 2 types of tasks, *P* < 0.0031 after Bonferroni correction). Notably, the components of decision uncertainty associated with task difficulty and RT were also represented in the dACC (S6B Fig). Thus, the dACC uniformly represented all components of decision uncertainty in metacognition.

### Common neural representations of estimate uncertainty across the mentalizing tasks

**Fig 5:**
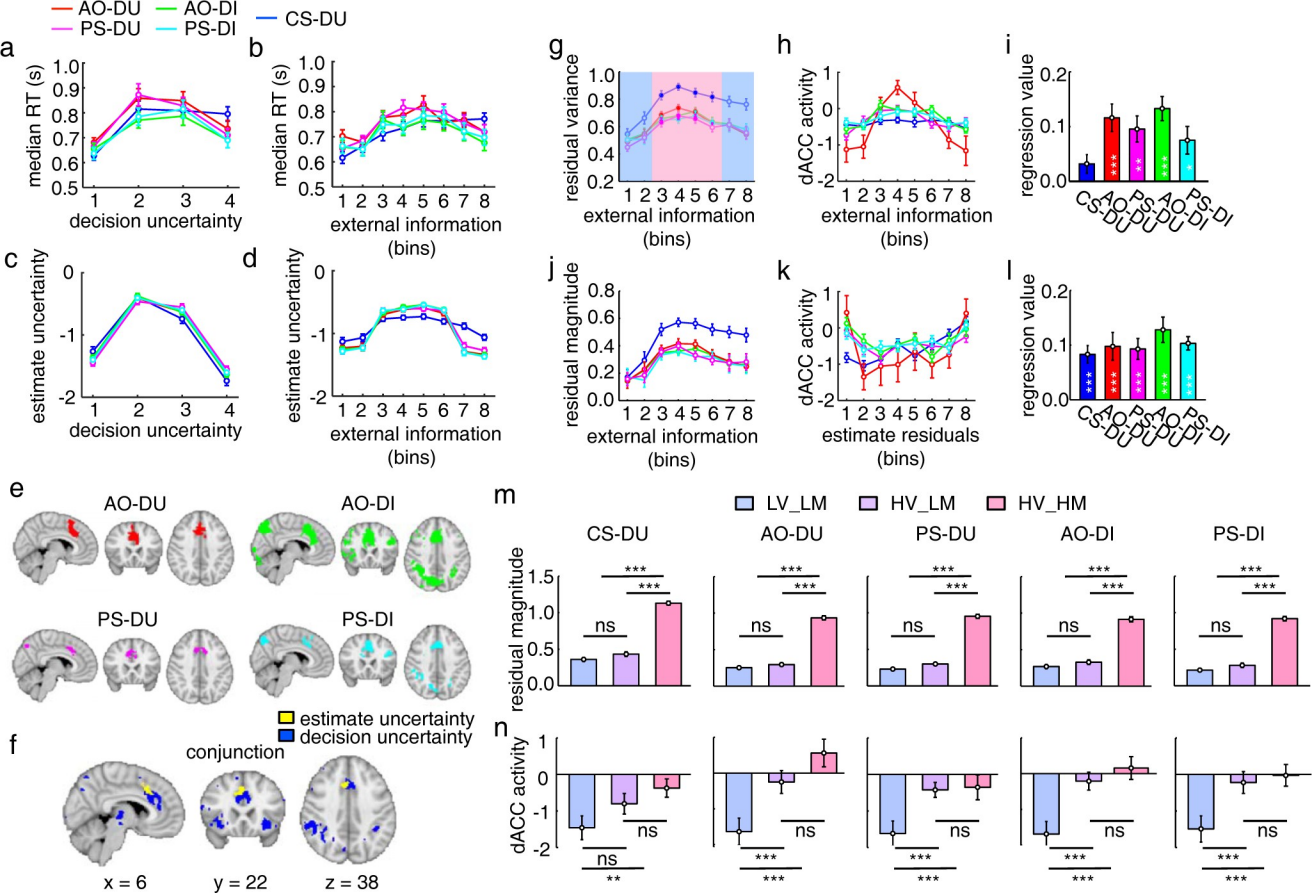
Common neural representations of estimate uncertainty across the mentalizing tasks. (**a**) The inverted U-shape relationships between RTs for reporting the estimates and the estimates commonly in the mentalizing tasks; (**b**) The inverted U-shape relationships between RTs for reporting the estimates and external information calculated by a sigmoid function of task difficulty and RT. (**c**) estimate uncertainty defined as (–1*|estimate–mean(estimates)|) had an inverted U-shape relationship with the estimates. (**d**) Estimate uncertainty had an inverted U-shape relationship with external information. (**e**) The neural activities were significantly correlated with estimate uncertainty in each mentalizing task. (**f**) The dACC was commonly correlated with estimate uncertainty (the conjunction analysis, yellow), overlapping with those correlated with decision uncertainty in the metacognition task (blue, see also Fig 4A). All the activation maps were displayed in radiological convention. (**g**) The inverted U-shape relationships between residual variances and external information. (**h**) The dACC activities had an inverted U-shape relationship with external information. (**i**) The linear regression beta values of the dACC activities with the residual variances. (**j**) The residual magnitudes were larger at the middle levels of external information. (**k**) The dACC activities were larger when the residual magnitudes were larger. (**l**) The linear regression beta values of the dACC activities with residual magnitudes. (**m**) The trials were divided into 3 subgroups with the same residual variances but different residual magnitudes (HV-HM: high_variance-high_magnitude versus HV_LM: high_variance-low_magnitude) and the same residual magnitudes but different residual variances (HV-LM versus LV_LM: low_variance-low_magnitude). (**n**) The dACC activities were significantly different between different residual variances, but not between different residual magnitudes. The error bars represent SEM across the participants. *ns*, not significant; *P < 0.05; **P < 0.01; ***P < 0.001, uncorrected. The raw data for Fig 5 can be found in the Supporting information as S1 Data. AO-DI, anonymous other decision inaccuracy; AO-DU, anonymous other decision uncertainty; CS-DU, current-self decision uncertainty; dACC, dorsal anterior cingulate cortex; PS-DI, past-self decision inaccuracy; PS-DU, past-self decision uncertainty; RT, response time; SEM, standard error of the mean.

In the mentalizing tasks, the use of external cue associations cannot provide sufficient information to estimate the target participants’ trial-by-trial decision inaccuracy/uncertainty, which should be also much affected by random neural processes characterized by each target participant’s unique internal noises (*σ*_1_ and *σ*_2_, Fig 2F and 2H). Thereby, the estimating processes in mentalizing are often accompanied by uncertainty about the estimates, namely estimate uncertainty [33,34]. Hence, the estimate theory predicts that estimate uncertainty should be higher when the estimates are at the middle levels as opposed to the lowest or the highest level (inverted U-shape). The levels of estimate uncertainty could be appreciated by the RTs used for reporting the estimates. Longer RTs indicate higher uncertainty in making estimates. The RTs were longer at the middle levels than at the lowest or highest level (inverted U-shape, quadratic regression, 2-tailed *t* test, *P*s < 0.001; Fig 5A). Further, we divided all trials equally into 8 bins according to the quantities of external information that were calculated by a sigmoid function of task difficulty and RT after fitting with the estimated decision inaccuracy/uncertainty (Eq 1 in Methods). The RTs in reporting the estimates were also longer at the middle bins than at the lower and higher bins (inverted U-shape, quadratic regression, 2-tailed *t* test, *P*s < 0.001; Fig 5B). For the sake of simplicity, we operationally defined the trial-by-trial estimate uncertainty as the negative value of the deviation from the mean of the estimates in each trial, i.e.,–|estimate-mean(estimates)|. By virtue of this definition, estimate uncertainty was higher at the middle levels of the estimates (Fig 5C) and the quantities of external information (Fig 5D). Notably, estimate uncertainty was statistically uncorrelated with the estimates per se in each task.

We then examined neural correlates of estimate uncertainty in each task. The neural correlates of estimate uncertainty in each mentalizing task were similar to those of decision uncertainty in metacognition (S7 Fig). Commonly across the mentalizing tasks, the fMRI activities in the dACC were significantly and positively correlated with estimate uncertainty (Fig 5E). Critically, the dACC region associated with estimate uncertainty across the mentalizing tasks was largely overlapping with the dACC region associated with decision uncertainty in the metacognition task (Fig 5F). Notably, this is complementary with prior findings that the fMRI activities in the ventromedial prefrontal cortex (vmPFC) and the posterior cingulate cortex (PCC) are responsive to confidence about the estimates of different properties of the presented stimuli [33].

### dACC was involved in monitoring the mentalizing processes

According to the estimation theory [33,34], estimate uncertainty is derived from the variance of estimate residuals (residual variance), which is larger in the middle range of the quantities of external information (Fig 2F). Consisting with this prediction, the residual variance in each bin of the quantities of external information was a negative parabolic function in each task (inverted U-shape, quadratic regression, 2-tailed *t* test, *P*s < 0.01; Fig 5G). Accordingly, the dACC activity averaged in each bin of the quantities of external information was also a negative parabolic function in each mentalizing task (inverted U-shape, quadratic regression, 2-tailed *t* test, *P*s < 0.05; Fig 5H), but not in the metacognition task (quadratic regression, 2-tailed *t* test, t_27_ = −1.9; *P* = 0.07). In each mentalizing task, the dACC activities increased as the residual variance increased (linear regression, 2-tailed *t* test, *P*s < 0.05, Fig 5I). Hence, the dACC activities tracked residual variances, suggesting its potential role in monitoring the mentalizing processes.

However, larger variances of estimate residuals might be accompanied by larger magnitudes of estimate residuals (residual magnitudes). To examine their concurrences, we calculated the mean residual magnitude averaged in each bin of the quantities of external information. Residual magnitudes had a similar relationship with the bins of external information as residual variances did (quadratic regression, 2-tailed *t* test, *P*s < 0.001; Fig 5J). Accordingly, the dACC activity in each bin according to the signed values of estimate residuals (the mean was zero) had a positive parabolic function in each of the mentalizing tasks, as well as in the metacognition task (U-shape, quadratic regression, 2-tailed *t* test, *P*s < 0.001; Fig 5K). Thus, the dACC activities also tracked residual magnitudes (linear regression, 2-tailed *t* test, *P*s < 0.001, Fig 5L).

As the dACC has been suggested to play a critical role in adaptive control [35–37], it remains possible for an alternative interpretation that the dACC might play a control role in adaptively generating estimate residuals. That is, the larger the dACC activities were, the larger residuals were generated. To distinguish the 2 potentially alternative functional roles of the dACC involvement in mentalizing, we further conducted partial control analyses. We segregated all trials into 3 categories according to residual variances and residual magnitudes. To do so, we first median split all trials according to residual variances (Fig 5G, low variances: blue zone, high variances: red zone). Accordingly, the residual magnitudes should be also low in the former group (LV_LM: low variances and low magnitudes). Further, the residual magnitudes in the latter group were segregated into low (HV_LM: high variances and low magnitudes) and high (HV_HM: high variances and high magnitudes) subgroups by another median split (Fig 5M). Thereby, the residual variances were different between LV_LM and HV_LM (2-tailed paired *t* test, *P*s < 6.7 × 10^−14^; Fig 5M), while their magnitudes were not different (2-tailed paired *t* test, *P*s > 0.05; Fig 5M). In contrast, the residual magnitudes were different between HV_LM and HV_HM (2-tailed paired *t* test, *P*s < 7.8 × 10^−13^; Fig 5M), while their variances were not different (2-tailed paired *t* test, *P*s > 0.18; Fig 5M). We then could test the 2 alternative hypotheses by examining whether the dACC activities were selectively responsive to residual variances (involvement in monitoring) or residual magnitudes (involvement in generating). If the dACC activities were sensitive to residual variances, but not residual magnitudes, then the dACC activities should be significantly different between LV_LM and HV_LM, but not between HV_LM and HV_HM. Otherwise, the dACC activity pattern should be reverse. Our results support the former prediction (Fig 5N). That is, the dACC should be involved in monitoring the process of generating internal mental states, rather than directly in the generating process. Hence, metacognition monitors the mentalizing processes.

**Fig 6:**
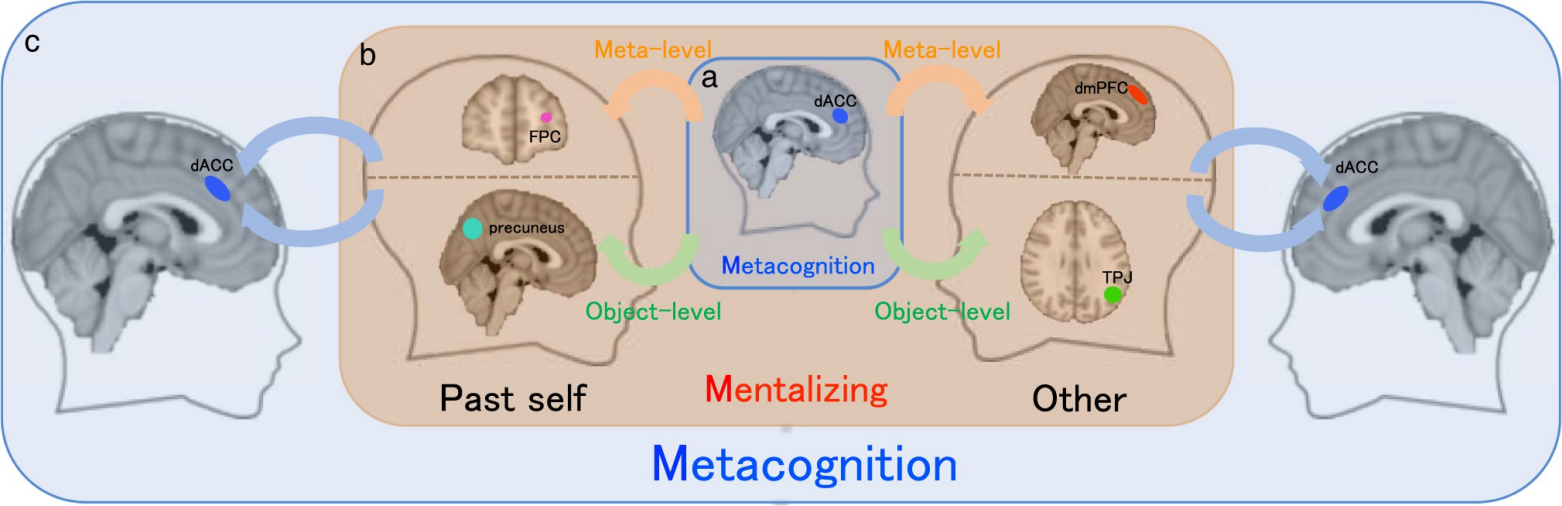
The nested structure of neural representations of decision uncertainty in metacognition and mentalizing. (**a**) The inner layer illustrates the neural representations of decision uncertainty in the dACC during monitoring one’s own decision-making by metacognition. (**b**) The middle layer illustrates distinct neural representations of decision uncertainty during monitoring the different target participants’ meta-level and object-level performance (left: the PS; right: others). The TPJ represents others’ decision inaccuracy (mental construct of the object-level performance) and the dmPFC represents others’ decision uncertainty (mental construct of the metacognitive performance). In contrast, the precuneus represents the PS-DI, and the lFPC represents the PS-DU. (**c**) The outer layer illustrates neural representations of estimate uncertainty in the dACC during the different mentalizing tasks, denoting that metacognition monitors mentalizing. dACC, dorsal anterior cingulate cortex; FPC, frontopolar cortex; dmPFC, dorsomedial prefrontal cortex; lFPC, lateral frontopolar cortex; PS, past self; PS-DI, past-self decision inaccuracy; PS-DU, past-self decision uncertainty; TPJ, temporoparietal junction.

Although estimate uncertainty and decision uncertainty have been suggested to be distinct [34], our results illustrate that the neural representations of decision uncertainty in metacognition (the inner layer in Fig 6) and estimate uncertainty in mentalizing (the outer layer in Fig 6) were convergingly registered in the dACC. On the contrary, mentalizing was functionally irrelevant to metacognition and was neurally independent of the dACC activities. Instead, different forms of mentalizing had diverse formats of internal mental state representations in the human brain (the middle layer in Fig 6). Mental state representations of decision uncertainty in metacognition and mentalizing form a nested structure (Fig 6).

## Discussion

Metacognition and mentalizing embody meta-level representations of mental states attributed to the observer and the target, respectively (Fig 1). However, the distinctions and relationship between metacognition and mentalizing are still a matter of debate [38]. In the current study, we adapted an experimental paradigm from metacognition to mentalizing. We could parametrically distinguish the neural representations of decision uncertainty between metacognition and mentalizing. Using fMRI to characterize the neural signals correlated with the corresponding mental states, we identified for the first time the different mental state representations underlying attributions of decision inaccuracy/uncertainty to different targets (the current self, the PS, and others), separated from the associations with external cue information. These separable internal mental state representations of decision uncertainty clearly underlie the distinctions between mentalizing from metacognition.

Metacognition in the current study was associated with the currently experienced decision uncertainty. Behaviorally, even after the perceptual information of task difficulty and RT was regressed out, estimate residuals still stably predicted actual decision inaccuracy, as the internal information about the neural processing information were accessible by introspection [1–3]. Neurally, the dACC unanimously represented these components to coherently form currently experienced decision uncertainty. In contrast, mentalizing in the current study was associated with inferred decision uncertainty. The participant could use external cue information to infer the covert mental states. Unsurprisingly, after the external cue information was regressed out, estimate residuals did not further predict the target participants’ actual internal mental states of decision uncertainty. Nonetheless, stable neural representations of estimate residuals were reliably observed in each of the mentalizing tasks. Critically, these neural representations of estimate residuals in attributing different levels (object level and meta level) of mental states to different targets (others and the PS) were distinct. These empirical results thus support the theoretical account: Mentalizing recruits additional mental processes of perspective taking beyond object-level associations with external cue information. Critically, these internal mental state representations made essential distinctions of mentalizing in different social contexts.

The type 1 mentalizing task in monitoring an AO’s decision inaccuracy was similar to false-belief tasks (e.g., the “Sally–Anne” task) [4,39,40]. Common to both tasks, the participant needs to judge the target participant’s object-level performance, i.e., the reversal of choice probability of the correct option (Sally should always choose the incorrect option in the “Sally–Anne” task), while the participant actually knows the ground truth. Thus, the participant’s mental world and the target participant’s mental world are different. To attribute the object-level cognitive states to the target participant, the participant might parsimoniously make a counterfactual inference on the basis of cue associations, which is though conflicted with the ground truth. To take the target participant’s perspective, the participant should merely build up a model describing the target participant’s task performance (type 1) with common knowledge that each person should have a unique capability in task performance (e.g., internal noise *σ*_1_) even under the same stimulus presentation. Our results imply that the TPJ activities might be associated with this mental process. This notion is consistent with prior evidence that the TPJ activations are prevalently observed in false-belief tasks [18,35,36], while anatomical and virtual lesions in the TPJ region selectively cause serious deficits in perspective taking for object-level performance evaluation [41,42].

The type 2 mentalizing task in monitoring an AO’s decision uncertainty was identical to the type 1 mentalizing task except that the participant instead estimated the target participant’s meta-level mental state of decision uncertainty. Hence, beyond the mental processes involved in the type 1 mentalizing task, the participant needed a mental model for the target participant’s metacognitive ability (Fig 2H). Our results imply that this mental model might be built in the dmPFC, which was selectively activated in the type 2 mentalizing task, but not in the type 1 mentalizing task. Although both the dmPFC and the TPJ have been shown to be involved in mentalizing [7,19,43], our findings implicate a putative functional distinction. The dmPFC was specifically involved in constructing the mental model of the target participant’s meta-level mental states [44,45]. In striking contrast, the TPJ might be involved in representing the target participant’s object-level mental states. These empirical results support the theoretical account of hierarchical neural representations of object-level and meta-level mental states in mentalizing. Intriguingly, when the identical task sequence was used but the target participant was changed to the PS, the neural loci associated with the internal mental states were altered. Thereby, these other-oriented neural representations of decision uncertainty in the dmPFC and TPJ regions support ToM in accounting for mentalizing [7,19,43].

When the participant estimated the PS-DI, the activities in the precuneus was selectively associated with estimate residuals. On the other hand, when the participant estimated the PS-DU, the activities in the lFPC were selectively associated with estimate residuals. The lFPC and precuneus regions are also both associated with the metacognitive process [15,16,32]. Hence, these results suggest that mentalizing for the PS mental states of decision uncertainty might recruit neural loci shared with metacognition. In other words, these self-oriented neural representations of decision uncertainty in the lFPC and the precuneus support the simulation theory accounting for mentalizing [46]. However, the dACC, the crucial brain region representing currently experienced decision uncertainty in metacognition [15–17,31], was not activated in the PS-oriented mentalizing tasks (S6B Fig).

When the participant monitored the target participants’ object-level performance in the type 1 mentalizing task, similar in monitoring the current-self object-level performance in the metacognition task, the estimates were actually the subjective beliefs of the participant, rather than the target participants. However, the neural representations of the mental states in the type 1 mentalizing tasks were entirely different from those in the metacognition task. To this end, a critical distinction between metacognition and mentalizing should depend on the accessibility of sources to be monitored, rather than the agents by whom the mental states are generated or the target participant to whom the mental states are attributed. Altogether, our results illustrate that the human brain diversifies separate neural systems to represent the different internal mental states of decision uncertainty in monitoring the current self, the PS, and others in performing the same perceptual decision-making task.

Importantly, the current neuroimaging results illustrate the relationship between metacognition and mentalizing, a longstanding puzzle in psychology and philosophy [38]. First, the mental state representations in metacognition and mentalizing are clearly dissociated. Metacognition and mentalizing are 2 independent processes with different meta-level mental state representations. Second, metacognition accompanies and monitors mentalizing, but mentalizing is independent of metacognition. Notably, mentalizing is a perception-based social inference process. Hence, metacognition as a domain-general process also monitors the high-level cognitive processes of mentalizing [15]. Last, even though the participants did not explicitly report estimate uncertainty during mentalizing, the dACC could implicitly embody estimate uncertainty [33].

Mentalizing is a crucial social cognitive function for human behaviors. During interpersonal interactions, the primary motivation of mentalizing is to predict and influence others’ beliefs, desires, and intentions, as well as their actions [45]. It is plausibly an effective strategy to manipulate influences on others when they are uncertain, rather than when they are highly confident, since the odds of success in changing others’ minds in the former case should be higher. Even for preverbal infants, when they feel uncertain, they are willing to seek caregiver’s helps [47]. Therefore, commonly in the mentalizing tasks, the target participants’ decision inaccuracy/uncertainty, rather than the reversals, decision accuracy/confidence, were predominantly and positively correlated with the brain activities. These positive neural signals might be used to guide appropriate social control [48,49]. In other words, the dACC involves in monitoring the current self–decision-making in metacognition and the dmPFC involves in monitoring others’ decision-making in mentalizing, driving cognitive control and social control, respectively. The dACC region here is anatomically in the sulcus of anterior cingulate cortex (ACC), while the dmPFC region is dorsally neighboring dACC [50]. Prior studies have also shown that a region in the gyrus of ACC (ACCg) or the perigenual ACC (pgACC), ventrally neighboring the dACC, is also involved in monitoring and predicting the others’ behaviors [51], but specifically in tracking the others’ motivational values [52].

Notably, estimates of others’ covert mental states and even those of the PS mental states were not predictable for their actual internal mental states or performance after the associations with external cue information were regressed out. These findings implicate that internal mental states are hard to predict, partially due to the fact that the target participants’ attributes in both object-level and meta-level performance were unknown to the participants in the current study. One potential approach to improve the predictability of mentalizing could be through social learning from interpersonal interactions [49], to construct more reliable mental models for a specific target’s mental world from more subtle social information and social experience [53–55]. Meanwhile, metacognition might facilitate such social learning processes by monitoring the mentalizing processes.

In conclusion, our findings of distinct mental state representations of decision uncertainty in mentalizing for different targets provide new insight on neural computations of the internal mental models in different mentalizing processes.

## Methods

### Participants

We recruited 28 healthy right-handed participants (22 females, age: 23.5 ± 1.5 years old) to take part in all 5 tasks across 3 fMRI experiments. Informed consent was obtained from each participant in accordance with a protocol approved by Beijing Normal University Research Ethics Committee (ICBIR_A_0091_004).

### Experiments

There were 3 fMRI experiments in the current study. In experiment 1, the participants carried out the metacognition task alone. In experiment 2, the participants inside the scanner observed the target participants (an AO or the PS) performing the metacognition task outside the scanner and estimated the target participants’ object-level performance, i.e., decision inaccuracy (AO-DI and PS-DI). The 2 tasks were then referred to as the type 1 mentalizing tasks. In experiment 3, the participants inside the scanner instead estimated the target participants’ meta-level performance, i.e., decision uncertainty (AO-DU and PS-DU). The 2 tasks were then referred to as the type 2 mentalizing tasks. Experiment 1 and experiment 3 were conducted in the same session, but experiment 2 was conducted in another session. The tasks in each session were randomly interleaved and were counterbalanced across the participants.

### Metacognition task

In an aperture with a radius of 3 degrees of visual angle, 300 white dots (radius: 0.08 degrees, density: 2.0%) were randomly displayed on a black background (RDM). The dots moved in different directions at a speed of 8.0 degrees/second. The movement of each dot lasted 3 frames. A subset of dots moved coherently in the same direction (left, up, right, or down), while the other dots moved in different random directions. The participant was required to discriminate the net motion direction and rate his/her uncertainty about the decision by pressing a corresponding button. The uncertainty ratings were the reversals of confidence ratings. The participants were instructed to rate decision uncertainty at 4 levels (1 to 4). Level 1 indicated a belief of high probability that the decision was correct. Level 4 indicated a belief of low probability that the decision was correct (see S1 Fig). That is, the decision was believed close to the chance level. The task difficulty was determined by the percentage of coherently moving dots, with 4 levels of task difficulty randomly mixed in the task. The easiest and hardest levels were fixed at a coherence of 1% and 30%, respectively, while the 2 middle levels were set for each participant to achieve accuracy of 50% and 80%, determined by a staircase procedure in a practice session conducted several days prior to the fMRI experiments [15,31].

### Mentalizing tasks

A pair of participants who had similar stimulus coherences at the 50% and 80% accuracy level in the practice session jointly took part in the task (the stimulus coherences at the 2 task difficulty levels used in the mentalizing tasks were the means of their stimulus coherences, respectively). One (the target participant) performed the metacognition task outside the scanner, and another (the observer participant) observed the target participant’s performance inside the scanner. The 2 computers separately presented the stimuli but were connected and synchronized by the local network following the TCP/IP protocol. Hence, they simultaneously performed their own tasks with the same sequence (see below) but in response to different stimuli and task requirements. To avoid eliciting the observer participant’s own decision uncertainty, the stimulus presented to her/him inside the scanner was noiseless: The randomly moving dots remained stationary, and only the coherently moving dots moved. While the target participant was responding, the observer participant saw a progress bar representing the elapsed time. However, the observer participant could not see the target participant’s choice and reported decision uncertainty. The observer participant then reported the estimate of the target participant’s decision inaccuracy/uncertainty. In the PS-DU and PS-DI mentalizing tasks, the target participant was replaced by the PS. That is, the observer participant observed RDM task performance previously done by himself/herself.

### Task sequence

The sequences of the metacognition and mentalizing tasks were almost identical. In the metacognition task, each trial started with a green cross cue to indicate that the task stimulus would be presented 1 second later. The stimulus was then presented for 2 seconds, and 4 options of the moving directions were presented. The participant made a choice within 2 seconds. After a choice was made, 4 ratings from 1 (most certain) to 4 (most uncertain) were presented, and the participant reported the rating by pressing the corresponding button within 2 seconds. The intertrial interval (ITI) was jittered uniformly between 2 seconds and 6 seconds. On average, each trial lasted for 9 seconds. During the mentalizing tasks, although the sequence was identical to the metacognition task performed outside the scanner, the participant watched the noiseless stimulus presentation and the progress bar indicating elapsed time for the target participant’s responses and then reported the estimate rating of the target participant’s decision inaccuracy/uncertainty. Each task was conducted for 2 consecutive runs, each consisting of sixty trials.

### Behavioral analyses

The reported ratings of decision inaccuracy/uncertainty in each of the metacognition and mentalizing tasks were associated with task difficulty and RT. We used a sigmoid function to characterize the relationship as follows:

$$e_{i}=\frac{1}{1+{exp}^{-(\beta_{1}D_{i}+\beta_{2}{RT}_{i}+\beta_{0})}}+\varepsilon_{i},$$ (1)

where *e*_*i*_ represents the estimate of decision inaccuracy/uncertainty at the trial *i*, *D*_*i*_ and *RT*_*i*_ represent the task difficulty and the RT at the trial *i*, respectively, *β*_1_ and *β*_2_ represent the corresponding weights, and *β*_0_ represents the constant bias, *ε*_*i*_ represents the estimate residual. Task difficulties and RT were separately normalized in each task for each participant. Further, for the mentalizing tasks, we also considered an autoregressive (AR) model to account for the associations of the estimates between the contiguous trials. However, the autocorrelation of the estimates between the contiguous trials in each mentalizing task was trivial, indicating that the estimates were not dependent on the history, but only on the current trial.

### fMRI parameters

All fMRI experiments were conducted using a 3-T Siemens Trio MRI system with a 12-channel head coil (Siemens, Germany). Functional images were acquired with a single-shot gradient echo T_2_* echo-planar imaging (EPI) sequence with volume repetition time of 2 seconds, echo time of 30 ms, slice thickness of 3.0 mm and in-plane resolution of 3.0 × 3.0 mm^2^ (field of view: 192 × 192 mm^2^; flip angle: 90 degrees). A total of 38 axial slices were taken with interleaved acquisition, parallel to the anterior commissure-posterior commissure line.

### fMRI analyses

fMRI analyses were conducted using FMRIB Software Library (FSL) [56]. To correct for rigid head motion, all EPI images were realigned to the first volume of the first scan. Data sets in which translation motion was larger than 2.0 mm or rotation motion was larger than 1.0 degree were discarded. No data were discarded in these analyses. Brain matter was separated from nonbrain using a mesh deformation approach and used to transform the EPI images to individual high-resolution structural images and then to the Montreal Neurological Institute (MNI) space by using affine registration with 12 degrees of freedom and resampling the data with a resolution of 2 × 2 × 2 mm^3^. Spatial smoothing with a 4-mm Gaussian kernel (full width at half-maximum) and high-pass temporal filtering with a cutoff of 0.005 Hz were applied to all fMRI data.

We used GLM to analyze the fMRI data. For the first-level analyses, 2 events were modeled in each trial. The first event (the perception phase) represented the stimulus presentation, time locked to the onset of the stimulus presentation, with a duration of the presentation time (2 seconds). The second event (the judgment phase) represented estimating decision inaccuracy/uncertainty, time locked to the onset of the rating, with the duration of the reaction time. The parametric modulation effects of task difficulty and RT, estimate residual, and estimate uncertainty were simultaneously added in the latter event (the judgment phase). Estimate residual was the residual of the estimate after the components associated with task difficulty and RT were regressed out. All the regressors were convolved with the canonical hemodynamic response function with double-gamma kennels. The results when the parametric modulation effects were added in the perception phase are presented in S3B Fig.

For the group-level analyses, we used FMRIB’s local analysis of mixed effects (FLAME), which models both “fixed effects” of within-participant variance and “random effects” of between-participant variance using Gaussian random-field theory in each task. Statistical parametric maps were generated with a threshold of *z* > 3.1, *P* < 0.05 after cluster-level FWE correction for multiple comparisons for each contrast, unless mentioned otherwise. The activation maps were displayed in radiological convention.

### Conjunction analyses

We conducted conjunction analyses to test the common neural correlates of external information (task difficulty and RT) across the 4 mentalizing tasks, as well as the common neural correlates of the estimate uncertainty across the 4 mentalizing tasks. We identified significant regions in which there was evidence of effects in all contrasts of conditions using the FSL script *easythresh*. Statistical parametric maps were generated by the threshold with *z* > 2.6, *P* < 0.05 after cluster-level FWE correction for each contrast.

### Region of interest (ROI) analyses

The IPL ROI was defined by the conjunction analysis across the 4 mentalizing tasks. The dACC ROI was defined in the CS-DU task. The dmPFC ROI was defined in the AO-DU task. The TPJ ROI was defined in the conjunction analysis between the AO-DU and AO-DI tasks. The FPC ROI was defined in the PS-DU task. The precuneus ROI was defined in the PS-DI task. To circumvent circular inference, we randomly analyzed the data from three-fourths of the participants to define each of the ROIs with a significance of z > 2.6, *P* < 0.05 after cluster-level FWE correction and then used the data from the held-out participants to obtain the parametric regression beta value in each ROI. We repeated this analysis one hundred times and averaged the parametric regression beta values for each ROI. For the dmPFC and TPJ ROIs, we further assessed meta-analytically derived ROIs associated with from Neurosynth (search term: “mentalizing”) [27], as well as the overlapping regions between our results and the Neurosynth ROIs. For the dACC region used for analyses in Fig 5, we used the overlap between the metacognition task and the mentalizing tasks obtained by conjunction analysis.

To identify whether the dACC activities in mentalizing tasks were associated with residual variances (i.e., in monitoring the mentalizing process) or residual magnitudes (i.e., in control of the mentalizing process), we sequentially divided the trials in each of the metacognition and mentalizing tasks into 3 subgroups according to the residual variances and the residual magnitudes. First, we divided all the trials equally into 8 bins according to the quantities of external information that were calculated by a sigmoid function of task difficulty and RT. We then split the bins of trials into 2 subgroups with low and high residual variances according to whether the residual variance in each bin was above the median and further split the trials with high residual variances into 2 subgroups with low and high residual magnitudes according to whether the residual magnitude of each trial was above the median (Fig 5J). We then calculated and compared the mean dACC activities among the 3 subgroups of trials (Fig 5K).

To test for the possibility of U-shaped relationships between dACC activity and estimate uncertainty, RT, or external information (Fig 5), we used a quadratic regression model

$$y=ax^{2}+bx+c$$ (2)

to fit with the data in each participant (polyfit in matlab) and tested the quadratic regression beta value (*a*) across the participants in comparison to zero using 2-tailed *t* test.

### Simulations and parameter recovery

Because the stimulus presentation and uncertainty rating events were temporally close to each other, the GLM estimates for the 2 events should be highly collinear. To examine whether the GLM analyses could separate the underlying neural activity specifically associated with each of the 2 events, we generated fMRI data using the different parameters of the GLM as follows and validated the parameter recovery [29].

$$y_{fMRI}=(\beta_{1}+\beta_{2}\times DU)\times X_{sti}⊗hrf+(\beta_{3}+\beta_{4}\times DU)\times X_{rating}⊗hrf+ℇ,$$ (3)

where X_sti_ and X_rating_ represent the design matrix for the perception phase and the judgment phase, respectively. *β*_1_ and *β*_3_ are the mean activities of the 2 events, and *β*_2_ and *β*_4_ are the estimate uncertainty modulation effects on the 2 events, respectively. *hrf* is the canonical hemodynamic response function with 2-gamma kennels, and ℇ is an additional Gaussian noise. The values of *β*_1_ and *β*_3_, as well as *β*_2_ or *β*_4_, were independently and randomly drawn from a uniform distribution in the range of 0.2 to 0.8, while the alternative *β*_2_ or *β*_4_ was set to zero. That is, the modulation effect appeared only in either of the 2 phases, but not simultaneously in both phases. The signal-to-noise ratio (SNR) of the fMRI data were set uniformly in the range of 0.01 to 1 (the event-evoked fMRI signals are usually in the range of 0.1 to 10 of the noises). We then used the GLM with the mean activity and the parametric modulation effects in both phases to reconstruct the mean activity and the parametric modulation effects in the 2 events from the generated fMRI data with the sampling rate of 0.5 Hz (TR = 2 seconds). For each set of the parameters, 1,000 times of procedures were repeated, and the estimated values were averaged at each SNR level (S3 Fig).

## Supporting information

S1 Fig The description of the instructions of tasks. (EPS)

S2 Fig Performance accuracy, decision uncertainty, and RT changed with task difficulty in the RDM task. (**a**) Performance accuracy and RT changed with task difficulty. (**b**) Decision uncertainty changed with task difficulty in the correct and incorrect trials, respectively. (**c**) RT changed with task difficulty in the correct and incorrect trials. The 4 task difficulty levels for each individual participant were set at 1% coherence, 50% accuracy (actually 55% inside the scanner), 80% accuracy, and 30% coherence, respectively (chance accuracy level: 25%). The error bars represent SEM across participants. The raw data for S2 Fig can be found in the Supporting information as S1 Data. RDM, random dots motion; RT, response time; SEM, standard error of the mean.
(EPS)

S3 Fig Reliable separation of neural activity during the perception phase and the judgment phase. The fMRI signals were simulated by the GLM that took into account of the separate contributions from the 2 phases, where DU represents the modulation effect of decision uncertainty, Ɛ represents the Gaussian noise, and hrf represents the canonical hemodynamic response function. The fMRI signals were obtained by the GLM with different mean values (0.2 to 0.8) simultaneously at both phases and the decision uncertainty modulation component (0.2 to 0.8) was added into the perception phase or the judgment phase. However, the recovered parameters were fitted by the GLM with the parametric modulation effects in both phases. For each set of parameters, procedures were repeated 1,000 times, and the estimated values were averaged at each SNR level. (**a**) The correlation between the components of generated fMRI time series in the perception phase and in the judgment phase in each participant (the event sequence was identical to the AO-DU task). (**b**) The ratio of the recovered mean activity (*β*_1_ and *β*_3_) to the original mean activity in each corresponding phase under the different SNR levels. (**c**) The recovered parametric modulation effects (*β*_2_ in the perception phase and *β*_4_ in the judgment phase) under the different SNR levels. The original mean activities were in both phases but the parametric modulation effect was in the stimulation phase in (**b**) and (**c**). (**d**) The ratio of the recovered mean activity (*β*_1_ and *β*_3_) to the original mean activity in each corresponding phase under the different SNR levels. (**e**) The recovered parametric modulation effects (*β*_2_ in the perception n phase and *β*_4_ in the judgment phase) under the different SNR levels. The original mean activities were in both phases but the parametric modulation effect was in the judgment phase in (**d**) and (**e**). (**f–i**) The parametric regression beta values in the GLM for the modulation effects at the perception phase for the empirical data. Except for the dACC in the CS-DU task, the modulation effects were not significant in the other tasks or in the other ROIs. The error bars represent SEM across participants. **P < 0.01, after Bonferroni correction. The raw data for S3 Fig can be found in the Supporting information as S1 Data. AO-DU, anonymous other decision uncertainty; CS-DU, current-self decision uncertainty; dACC, dorsal anterior cingulate cortex; fMRI, functional magnetic resonance imaging; GLM, general linear model; ROI, region of interest; SEM, standard error of the mean; SNR, signal-to-noise ratio.
(EPS)

S4 Fig Relationships between the weights of task difficulty and RT in accounting for the estimates of decision inaccuracy/uncertainty across tasks. The weights of task difficulty and RT were highly correlated and equivalent (falling along the diagonal) across the mentalizing tasks. However, the weights of task difficulty were larger in the mentalizing tasks than in the metacognition task, but the weights of the RTs were reversed. The raw data for S4 Fig can be found in the Supporting information as S1 Data. RT, response time.
(EPS)

S5 Fig The parametric regression beta values of fMRI activity with estimate residuals in each task in the meta-analytically derived ROIs. (**a**) The meta-analytically derived ROIs associated with “mentalizing” from Neurosynth. (**b**) The overlapped regions between our results and the meta-analytically derived ROIs. The activation maps were displayed in radiological convention. The convention of the colors is the same as in Fig 4. The error bars represent SEM across participants. *ns*, not significant; *P < 0.05; **P < 0.01; ***P < 0.001, uncorrected. The raw data for S5 Fig can be found in the Supporting information as S1 Data. fMRI, functional magnetic resonance imaging; ROI, region of interest; SEM, standard error of the mean.
(EPS)

S6 Fig The parametric beta values of fMRI activity regressed with task difficulty, RT, and estimate residuals in each task in the ROIs. (**a**) IPL. (**b**) dACC. (**c**) TPJ. (**d**) dmPFC. (**e**) precuneus. (**f**) FPC. The convention of the colors is the same as in Fig 4. The error bars represent SEM across participants. *P < 0.05; **P < 0.01; ***P < 0.001, uncorrected. The raw data for S6 Fig can be found in the Supporting information as S1 Data. dACC, dorsal anterior cingulate cortex; dmPFC, dorsomedial prefrontal cortex; fMRI, functional magnetic resonance imaging; FPC, frontopolar cortex; IPL, inferior parietal lobe; ROI, region of interest; RT, response time; SEM, standard error of the mean; TPJ, temporoparietal junction.
(EPS)

S7 Fig Activation maps showing significantly correlated with estimate uncertainty in each task. (**a**) CS-DU. (**b**) AO-DU. (**c**) PS-DU. (**d**) AO-DI. (**e**) PS-DI. *z* > 3.1, *P* < 0.05, after cluster-level FWE correction. AO-DI, anonymous other decision inaccuracy; AO-DU, anonymous other decision uncertainty; CS-DU, current-self decision uncertainty; FWE, family-wise error; PS-DI, past-self decision inaccuracy; PS-DU, past-self decision uncertainty.
(EPS)

S1 Table Summary of brain activations. (DOCX)

S1 Data Raw data for all figures. (XLSX)

## Abbreviations

ACC: anterior cingulate cortex
ACCg: gyrus of ACC
ANOVA: analysis of variance
AO: anonymous other
AO-DI: anonymous other decision inaccuracy
AR: autoregressive
ASD: autism spectrum disorder
AUROC: area under the ROC curve
CS-DU: current-self decision uncertainty
dACC: dorsal anterior cingulate cortex
dmPFC: dorsomedial prefrontal cortex
EPI: echo-planar imaging
FLAME: FMRIB’s local analysis of mixed effects
fMRI: functional magnetic resonance imaging
FSL: FMRIB Software Library
FWE: family-wise error
GLM: general linear model
IFJ: inferior frontal junction
IPL: inferior parietal lobe
ITI: intertrial interval
lFPC: lateral frontopolar cortex
MNI: Montreal Neurological Institute
PCC: posterior cingulate cortex
pgACC: perigenual ACC
PS: past self
PS-DI: past-self decision inaccuracy
PS-DU: past-self decision uncertainty
RDM: random dots motion
ROC: receiver operating characteristic
ROI: region of interest
RT: response time
SNR: signal-to-noise ratio
ToM: theory of mind
TPJ: temporoparietal junction
vmPFC: ventromedial prefrontal cortex

## References

[1] Perner J. Understanding the representational mind. The MIT Press; 1991.

[2] Suddendorf T, Whiten A. Mental evolution and development: Evidence for secondary representation in children, great apes, and other animals. Psychol Bull. 2001;127(5):629–50. doi: 10.1037/0033-2909.127.5.629 11548971

[3] Flavell JH. Cognitive development: Children’s knowledge about the mind. Annu Rev Psychol. 1999;50(1):21–45. doi: 10.1146/annurev.psych.50.1.21 10074674

[4] Baron-Cohen S, Leslie AM, Frith U. Does the autistic child have a “theory of mind”? Cognition. 1985;21(1):37–46. doi: 10.1016/0010-0277(85)90022-8 2934210

[5] Frith CD. The cognitive neuropsychology of schizophrenia. Psychology Press; 1992.

[6] Flavell JH. Metacognition and cognitive monitoring: A new area of cognitive–developmental inquiry. Am Psychol. 1979;34(10):906–91.

[7] Frith CD, Frith U. Mechanisms of social cognition. Annu Rev Psychol. 2012;63:287–313. doi: 10.1146/annurev-psych-120710-100449 21838544

[8] Zaki J, Ochsner K. You, me, and my brain: Self and other representations in social cognitive neuroscience. In: Todorov A, Fiske ST, Prentice DA, editors. Social neuroscience: Toward understanding the underpinnings of the social mind: Oxford University Press; 2011. p. 14–39.

[9] Pierce JL, Kostova T, Dirks KT. The state of psychological ownership: Integrating and extending a century of research. Rev Gen Psychol. 2003;7(1):84–107.

[10] Nelson TO. Metamemory: A theoretical framework and new findings. Psychology of learning and motivation. Psychol Learn Motiv. 1990;26:125–73.

[11] Premack D, Woodruff G. Does the chimpanzee have a theory of mind? Behav Brain Sci. 1978;1(4):515–26.

[12] Gopnik A, Wellman HM. Why the child’s theory of mind really is a theory. Mind Lang. 1992;7:145–71.

[13] Fodor JA. The modularity of mind. MIT Press; 1983.

[14] Wimmer H, Perner J. Beliefs about beliefs: Representation and constraining function of wrong beliefs in young children’s understanding of deception. Cognition. 1983;13(1):103–28. doi: 10.1016/0010-0277(83)90004-5 6681741

[15] Qiu L, Su J, Ni Y, Bai Y, Zhang X, Li X, et al. The neural system of metacognition accompanying decision-making in the prefrontal cortex. PLoS Biol. 2018;16(4):e2004037. doi: 10.1371/journal.pbio.2004037 29684004 PMC5933819

[16] Fleming SM, Huijgen J, Dolan RJ. Prefrontal contributions to metacognition in perceptual decision making. J Neurosci. 2012;32(18):6117–25. doi: 10.1523/JNEUROSCI.6489-11.2012 22553018 PMC3359781

[17] Pereira M, Faivre N, Iturrate I, Wirthlin M, Serafini L, Martin S, Desvachez A, Blanke O, Van De Ville D, Millan J del R. Disentangling the origins of confidence in speeded perceptual judgments through multimodal imaging. Proc Natl Acad Sci U S A. 2020;117:8382–8390. doi: 10.1073/pnas.1918335117 32238562 PMC7165419

[18] Bernhardt BC, Singer T. The neural basis of empathy. Annu Rev Neurosci. 2012;35:1–23. doi: 10.1146/annurev-neuro-062111-150536 22715878

[19] Schurz M, Radua J, Aichhorn M, Richlan F, Perner J. Fractionating theory of mind: a meta-analysis of functional brain imaging studies. Neurosci Biobehav Rev. 2014;42:9–34. doi: 10.1016/j.neubiorev.2014.01.009 24486722

[20] Wan X, Cheng K, Tanaka K. The neural system of postdecision evaluation in rostral frontal cortex during problem-solving tasks. eNeuro. 2016;3(4):e0188–16. doi: 10.1523/ENEURO.0188-16.2016 27595134 PMC5002985

[21] Bahrami B, Olsen K, Latham PE, Roepstorff A, Rees G, Frith CD. Optimally interacting minds. Science. 2010;329(5995):1081–5. doi: 10.1126/science.1185718 20798320 PMC3371582

[22] Koriat A. When are two heads better than one and why? Science. 2012;336(6079):360–2. doi: 10.1126/science.1216549 22517862

[23] Baron-Cohen S, Wheelwright S, Hill J, Raste Y, Plumb I. The “Reading the Mind in the Eyes” test revised version: A study with normal adults, and adults with Asperger syndrome or high-functioning autism. J Child Psychol Psychiatry. 2001;42(2):241–51. 11280420

[24] Kliemann D, Adolphs R. The social neuroscience of mentalizing: challenges and recommendations. Curr Opin Psychol. 2018;24:1–6. doi: 10.1016/j.copsyc.2018.02.015 29529497 PMC6110997

[25] Lurz RW. Mindreading animals: The debate over what animals know about other minds. MIT Press; 2011.

[26] Heyes C. Apes submentalise Trends Cogn Sci. 2017;21:1–2. doi: 10.1016/j.tics.2016.11.006 27919697

[27] Pleskac TJ, Busemeyer JR. Two-stage dynamic signal detection: a theory of choice, decision time, and confidence. Psychol Rev. 2010;117(3):864–901. doi: 10.1037/a0019737 20658856

[28] Fleming SM, Weil RS, Nagy Z, Dolan RJ, Rees G. Relating introspective accuracy to individual differences in brain structure. Science. 2010;329(5998):1541–3. doi: 10.1126/science.1191883 20847276 PMC3173849

[29] Bang D, Fleming SM. Distinct encoding of decision confidence in human medial prefrontal cortex. Proc Natl Acad Sci U S A. 2018;115(23):6082–7. doi: 10.1073/pnas.1800795115 29784814 PMC6003322

[30] Yarkoni T, Poldrack RA, Nichols TE, Van Essen DC, Wager TD. Large-scale automated synthesis of human functional neuroimaging data. Nat Methods. 2011;8(8):665–70. doi: 10.1038/nmeth.1635 21706013 PMC3146590

[31] Jia W, Zhu H, Ni Y, Su J, Xu R, Jia H, et al. Disruptions of frontoparietal control network and default mode network linking the metacognitive deficits with clinical symptoms in schizophrenia. Hum Brain Mapp. 2020;41(6):1445–58. doi: 10.1002/hbm.24887 31789478 PMC7267896

[32] Vaccaro AG, Fleming SM. Thinking about thinking: A coordinate-based meta-analysis of neuroimaging studies of metacognitive judgements. Brain Neurosci Adv. 2018;2:239821281881059. doi: 10.1177/2398212818810591 30542659 PMC6238228

[33] Lebreton M, Abitbol R, Daunizeau J, Pessiglione M. Automatic integration of confidence in the brain valuation signal. Nat Neurosci. 2015;18(8):1159–67. doi: 10.1038/nn.4064 26192748

[34] Pouget A, Drugowitsch J, Kepecs A. Confidence and certainty: distinct probabilistic quantities for different goals. Nat Neurosci. 2016;19(3):366–74. doi: 10.1038/nn.4240 26906503 PMC5378479

[35] Behrens TE, Woolrich MW, Walton ME, Rushworth MF. Learning the value of information in an uncertain world. Nat Neurosci. 2007;10(9):1214–21. doi: 10.1038/nn1954 17676057

[36] Shenhav A, Botvinick MM, Cohen JD. The expected value of control: an integrative theory of anterior cingulate cortex function. Neuron. 2013;79(2):217–40. doi: 10.1016/j.neuron.2013.07.007 23889930 PMC3767969

[37] Kolling N, Wittmann MK, Behrens TE, Boorman ED, Mars RB, Rushworth MF. Value, search, persistence and model updating in anterior cingulate cortex. Nat Neurosci. 2016;19(10):1280–5. doi: 10.1038/nn.4382 27669988 PMC7116891

[38] Carruthers P. How we know our own minds: The relationship between mindreading and metacognition. Behav Brain Sci. 2009;32(2):121–82. doi: 10.1017/S0140525X09000545 19386144

[39] Saxe R, Kanwisher N. People thinking about thinking people: the role of the temporo-parietal junction in “theory of mind”. Neuroimage. 2003;19(4):1835–42. doi: 10.1016/s1053-8119(03)00230-1 12948738

[40] Saxe R, Wexler A. Making sense of another mind: the role of the right temporo-parietal junction. Neuropsychologia. 2005;43(10):1391–9. doi: 10.1016/j.neuropsychologia.2005.02.013 15936784

[41] Samson D, Apperly IA, Chiavarino C, Humphreys GW. Left temporoparietal junction is necessary for representing someone else’s belief. Nat Neurosci. 2004;7(5):499–500. doi: 10.1038/nn1223 15077111

[42] Soutschek A, Ruff CC, Strombach T, Kalenscher T, Tobler PN. Brain stimulation reveals crucial role of overcoming self-centeredness in self-control. Sci Adv. 2016;2(10):e1600992. doi: 10.1126/sciadv.1600992 27774513 PMC5072183

[43] Amodio DM, Frith CD. Meeting of minds: the medial frontal cortex and social cognition. Nat Rev Neurosci. 2006;7(4):268–77. doi: 10.1038/nrn1884 16552413

[44] Mitchell JP, Macrae CN, Banaji MR. Dissociable medial prefrontal contributions to judgments of similar and dissimilar others. Neuron. 2006;50(4):655–63. doi: 10.1016/j.neuron.2006.03.040 16701214

[45] Jenkins AC, Mitchell JP. How has cognitive neuroscience contributed to social psychological theory? In: Todorov A, Fiske ST, Prentice DA, editors. Social Neuroscience: Toward understanding the underpinnings of the social mind: Oxford University press; 2011. p. 3–13.

[46] Goldman AI. Simulating minds: The philosophy, psychology, and neuroscience of mindreading. Oxford University Press on Demand; 2006.

[47] Goupil L, Kouider S. Behavioral and neural indices of metacognitive sensitivity in preverbal infants. Curr Biol. 2016;26(22):3038–45. doi: 10.1016/j.cub.2016.09.004 27773566 PMC5130696

[48] van Schie HT, Mars RB, Coles MG, Bekkering H. Modulation of activity in medial frontal and motor cortices during error observation. Nat Neurosci. 2004;7(5):549–54. doi: 10.1038/nn1239 15107858

[49] Yoshida K, Saito N, Iriki A, Isoda M. Social error monitoring in macaque frontal cortex. Nat Neurosci. 2012;15(9):1307–12. doi: 10.1038/nn.3180 22864610

[50] Jamali M, Grannan BL, Fedorenko E, Saxe R, Báez-Mendoza R, Williams ZM. Single-neuronal predictions of others’ beliefs in humans. Nature. 2021;591(7851):610–4. doi: 10.1038/s41586-021-03184-0 33505022 PMC7990696

[51] Behrens TE, Hunt LT, Woolrich MW, Rushworth MF. Associative learning of social value. Nature. 2008;456(7219):245–9. doi: 10.1038/nature07538 19005555 PMC2605577

[52] Apps MA, Rushworth MF, Chang SW. The anterior cingulate gyrus and social cognition: tracking the motivation of others. Neuron. 2016;90(4):692–707. doi: 10.1016/j.neuron.2016.04.018 27196973 PMC4885021

[53] Rabinowitz N, Perbet F, Song F, Zhang C, Eslami SA, Botvinick M, editors. Machine theory of mind. Proceedings of the 35th International Conference on Machine Learning; 2018: PMLR.

[54] Baker CL, Jara-Ettinger J, Saxe R, Tenenbaum JB. Rational quantitative attribution of beliefs, desires and percepts in human mentalizing. Nat Hum Behav. 2017;1(4):1–10.

[55] Yoshida W, Dolan RJ, Friston KJ. Game theory of mind. PLoS Comput Biol. 2008;4(12):e1000254. doi: 10.1371/journal.pcbi.1000254 19112488 PMC2596313

[56] Smith SM, Jenkinson M, Woolrich MW, Beckmann CF, Behrens TE, Johansen-Berg H, et al. Advances in functional and structural MR image analysis and implementation as FSL. Neuroimage. 2004;23:S208–S19. doi: 10.1016/j.neuroimage.2004.07.051 15501092

