# Supplementary material for: Metacognition and mentalizing are associated with distinct neural representations of decision uncertainty
Source: PLoS Biol. 2022 May 13;20(5):e3001301. doi: 10.1371/journal.pbio.3001301 (PMC9132335; doi:10.1371/journal.pbio.3001301)
Supplement: S1 Table — (DOCX) [file pbio.3001301.s008.docx]

S1 Table. Summaries of brain activations

| **Variables** | | **Activated Region** | | | | **Hemi-**  **spheres** | **MNI Coordinates**  **(x, y, z)** | | | **Maximum** | | | |
| --- | --- | --- | --- | --- | --- | --- | --- | --- | --- | --- | --- | --- | --- |
| **CS-DU (RDM)** | | | | | | | | | | | | | |
| Task difficulty  (positive) | | Dorsal anterior cingulate cortex  (dACC, BA24) | | | | – | –4, 24, 36 | | | 4.40 | | | |
|  |  | Superior parietal lobule (BA7) | | | | L | –18, –70, 50 | | | 4.03 | | | |
|  |  | Frontopolar cortex  (FPC, BA10) | | | | L | –26, 50, 10 | | | 3.74 | | | |
|  |  | Insular cortex (BA47) | | | | L | –40, 22, –8 | | | 4.04 | | | |
| Task  Difficulty  (negative) | | Inferior parietal lobule  (IPL, BA40) | | | | L | –55, –44, 30 | | | –4.31 | | | |
|  |  | Middle temporal gyrus (BA37) | | | | L  R | –58, –52, –8  58, –42, –14 | | | –4.53  –4.20 | | | |
|  |  | Posterior cingulate cortex  (PCC, BA23) | | | | – | –6, –34, 40 | | | –4.29 | | | |
| Task difficulty  (negative) | | Middle temporal gyrus (BA37) | | | | L | –56, –50, –10 | | | -4.44 | | | |
| Response Time  (positive) | | Superior parietal lobule (BA7) | | | | L  R | –18, –70, 50  34, –54, 52 | | | 6.57  5.43 | | | |
|  |  | Dorsal anterior cingulate cortex  (dACC, BA24) | | | | – | 0, 10, 44 | | | 6.05 | | | |
|  |  | Insular cortex (BA47) | | | | L  R | –34, 20, –2  38, 20, 2 | | | 6.07  5.80 | | | |
| Response Time  (negative) | | Ventral medial prefrontal cortex  (vmPFC, BA11) | | | | – | –5, 40, –17 | | | -5.18 | | | |
| Decision  Uncertainty  (positive) | | Frontopolar cortex (BA10) | | | | R  L | 40, 58, 0  –26, 52, 6 | | | 4.71  4.12 | | | |
|  |  | Anterior cingulate cortex  (ACC, BA24) | | | | – | 4, 26, 30 | | | 4.97 | | | |
|  |  | Inferior parietal lobule (BA40) | | | | R | 46, –54, 46 | | | 3.98 | | | |
|  |  | Insular Cortex (BA47) | | | | L  R | –32, 20, –8  36, 20, –2 | | | 4.57  3.68 | | | |
| Decision  Uncertainty  (negative) | | Ventral medial prefrontal cortex (vmPFC, BA11) | | | | L | –12, 48, –16 | | | –4.13 | | | |
| 2^nd^ Decision Uncertainty  (negative) | | Posterior cingulate cortex  (PCC, BA23) | | | | – | 0, –42, 40 | | | | | –4.50 | |
|  |  | Angular gyrus  (BA39) | | | | R | 56, –54, 40 | | | | | –4.49 | |
|  |  | Ventral medial prefrontal cortex (BA10) | | | | – | 4, 44, –8 | | | | | –3.77 | |
| **AO-DU** | | | | | | | | | | | | | |
| Task  Difficulty  (positive) | | Superior parietal lobule  (BA7) | | | | R  L | 48, –40, 48  –28, –58, 34 | | | | | 4.45  4.36 | |
|  |  | Inferior frontal junction  (IFJ, BA44) | | | | R  L | 42, 16, 38  –46, 12, 34 | | | | | 4.46  4.42 | |
|  |  | Dorsal anterior cingulate cortex  (dACC, BA32) | | | | – | 2, 28, 44 | | | | | 3.83 | |
| Response Time  (positive) | | Superior parietal lobule (BA7) | | | | R  L | 28, –64, 48  –20, –66, 48 | | | | | 5.89  5.32 | |
|  |  | Occipital fusiform  (BA18) | | | | R  L | 26, –74, -17  –26, –75, -15 | | | | | 5.32  5.11 | |
| Decision  Uncertainty  (positive) | | Dorsomedial prefrontal cortex  (dmPFC, BA32) | | | | – | 0, 54, 20 | | | | | 3.84 | |
|  |  | Inferior frontal junction  (IFJ, BA48) | | | | L | –40, 13, 26 | | | | | 3.52 | |
|  |  | Temporoparietal junction  (TPJ, BA37) | | | | L | –58, –60, 18 | | | | | 4.33 | |
| Estimate Uncertainty  (positive) | | Dorsal anterior cingulate cortex  (dACC, BA24) | | | | R | 10, 18, 38 | | | | | 4.3 | |
|  |  | Inferior frontal junction  (IFJ, BA44) | | | | R | 52, 10, 14 | | | | | 3.8 | |
| Estimate Uncertainty  (negative) | | Posterior cingulate cortex  (PCC, BA23) | | | | – | –2, –48, 39 | | | | | -4.48 | |
|  |  | Temporoparietal junction  (TPJ, BA37) | | | | L | –52, –66, 14 | | | | | -4.29 | |
| **PS-DU** | | | | | | | | | | | | | |
| Task Difficulty  (positive) | | Superior parietal lobule (BA7) | | | | R | | | 30, –68, 48 | | | 3.92 | |
| Response Time  (positive) | | Superior parietal lobule (BA7) | | | | R  L | | | 32, –60, 52  –28, –60, 42 | | | 5.22  5.04 | |
|  |  | Occipital fusiform  (V4, BA18) | | | | R  L | | | 32, –86, 10  –26, –83, –17 | | | 6.07  5.70 | |
| Decision  Uncertainty  (positive) | | Frontopolar cortex  (FPC, BA10) | | | | L | | | –26, 54, 6 | | | 3.55 | |
| Estimate Uncertainty  (positive) | | Dorsal anterior cingulate cortex  (dACC, BA24) | | | | – | | | 2, 20, 40 | | | 3.96 | |
| **AO-DI** | | | | | | | | | | | | | |
| Task  Difficulty  (positive) | | Superior parietal lobule (BA7) | | R | | | | | 24, –78, 50 | | | 4.41 | |
| Task  Difficulty  (negative) | | Occipital cortex  (V1, BA17) | | R  L | | | | | 28, –96, –6  –16, –100, 6 | | | -5.15  -4.62 | |
| Response Time  (positive) | | Superior parietal lobule (BA7) | | R  L | | | | | 33, –75, 42  –22, –68, 44 | | | 4.47  4.55 | |
| Decision  Uncertainty  (positive) | | Inferior frontal junction  (IFJ, BA48) | | L | | | | | –48, 14, 30 | | | 3.92 | |
|  |  | Temporoparietal junction  (TPJ, BA39) | | L | | | | | –44, –62, 26 | | | 3.85 | |
| Estimate Uncertainty  (positive) | | Precuneus (BA7) | | R | | | | | 10, -72, 48 | | | 4.88 | |
|  |  | Dorsal anterior cingulate cortex  (dACC, BA24) | | – | | | | | –2, 10, 48 | | | 5.51 | |
|  |  | Inferior frontal junction  (IFJ, BA48) | | R  L | | | | | 52, 24, 26  –46, 28, 22 | | | 4.34  4.44 | |
|  |  | Frontopolar cortex  (FPC, BA11) | | R  L | | | | | 42, 56, 8  –26, 64, 4 | | | 4.04  4.68 | |
|  |  | Occipital cortex  (V1, BA17) | | R  L | | | | | 34, –94, –6  –18, –98, –6 | | | 4.31  4.27 | |
| **PS-DI** | | | | | | | | | | | | | |
| Task  Difficulty  (positive) | | Superior parietal lobule (BA7) | | R | | | | | 26, –62, 42 | | | 3.38 | |
| Task  Difficulty  (negative) | | Occipital cortex  (V1, BA18) | | R | | | | | 24, –92, –4 | | | –5.10 | |
| Response Time  (positive) | | Superior parietal lobule (BA7) | | R  L | | | | | 28, –62, 42  –26, –66, 46 | | | 4.80  4.15 | |
| Decision  Uncertainty  (positive) | | Precuneus (BA7) | | R | | | | | 4, –60, 50 | | | 4.09 | |
|  |  | Superior parietal lobule (BA7) | | L | | | | | –24, –66, 54 | | | 4.16 | |
|  |  | Dorsal lateral prefrontal cortex (dlPFC, BA44) | | L | | | | | –52, 14, 34 | | | 3.92 | |
| Estimate Uncertainty  (positive) | | Precuneus (BA7) | | R | | | | | 14, –68, 52 | | | 4.47 | |
|  |  | Dorsal anterior cingulate cortex  (dACC, BA32) | | – | | | | | 0, 18, 44 | | | 4.12 | |
| **Conjunction** | | | | | | | | | | | | | |
| Task  Difficulty  (positive) | | Superior parietal lobule (BA7) | | | | R | | | 30, –68, 48 | | | 3.88 | |
| Response Time  (positive) | | Superior parietal lobule (BA7) | | | | R  L | | | 30, –62, 42  –22, –68, 48 | | | 4.12  4.01 | |
|  |  | Occipital fusiform  (V4, BA18) | | | | L  R | | | –22, –78, –16  29, –74, –16 | | | 4.17  4.11 | |
| Estimate Uncertainty  (positive) | | Dorsal anterior cingulate cortex  (dACC, BA32) | | | | – | | | 2, 20, 42 | | | 3.82 | |
